# Supplementary material for: Engineered PW12-polyoxometalate docked Fe sites on CoFe hydroxide anode for durable seawater electrolysis
Source: Nat Commun. 2025 Jul 1;16:5541. doi: 10.1038/s41467-025-60620-9 (PMC12214576; doi:10.1038/s41467-025-60620-9)
Supplement: Supplementary file 1 — Supplementary Information [file 41467_2025_60620_MOESM1_ESM.pdf]

Supplementary Information

Engineered PW_{12}-polyoxometalate docked Fe sites on CoFe hydroxide anode for durable seawater electrolysis

Xun He[1,2,#], Yongchao Yao[1,3,#], Min Zhang[4], Yilei Zhou[5], Limei Zhang[3], Yuchun Ren[2], Kai Dong[4], Hong Tang[2], Jue Nan[2], Xingli Zhou[1], Han Luo[3], Binwu Ying[3], Qi Yu[5*], Fengming Luo[1*], Bo Tang[4,6*] & Xuping Sun[1,4*]

[1]Center for High Altitude Medicine, West China Hospital, Sichuan University, Chengdu 610041, Sichuan, China. [2]Institute of Fundamental and Frontier Sciences, University of Electronic Science and Technology of China, Chengdu 610054, Sichuan, China. [3]Department of Laboratory Medicine/Clinical Laboratory Medicine Research Center, West China Hospital, Sichuan University, Chengdu 610041, Sichuan, China. [4]College of Chemistry, Chemical Engineering and Materials Science, Shandong Normal University, Jinan 250014, Shandong, China. [5]School of Materials Science and Engineering, and Shaanxi Laboratory of Catalysis, Shaanxi University of Technology, Hanzhong 723001, Shaanxi, China. [6]Laoshan Laboratory, Qingdao 266237, Shandong, China. [#]Both authors contributed equally to this work.

[*]Correspondence and requests for materials should be addressed to Q.Y. (email: qiyu@snut.edu.cn) or F.L. (email: luofengming@wchscu.edu.cn) or B.T. (email: tangb@sdnu.edu.cn) or X.S. (email: xpsun@uestc.edu.cn).

Supplementary Note 1: Chloride ions accelerate electrode degradation through chemical corrosion. The degradation of electrode materials in chloride-rich media is governed by a mechanistically distinct leaching-corrosion pathway. The process initiates with the polarization-induced adsorption of Cl^- onto the electrode surface, forming metal-chloride intermediates. These intermediates subsequently interact with excess Cl^- to form soluble MCl_x^- species, representing a critical leaching step where active metal centers are removed from the solid phase. This dissolution weakens the structural framework of the catalyst. In the alkaline medium, the metal-chloride species undergo hydrolysis to yield metal hydroxides and release free Cl^-. These ions persist in the electrolyte, enabling recurrent corrosion cycles that progressively deteriorate electrode performance and longevity.

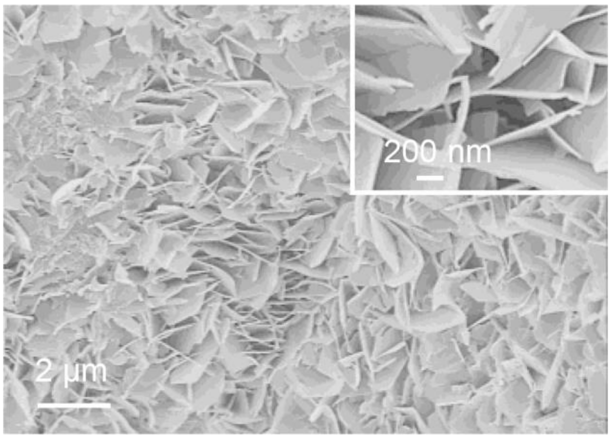

Supplementary Fig. 1 | SEM images. Low- and high-magnification SEM images of CoFe LDH/NF.

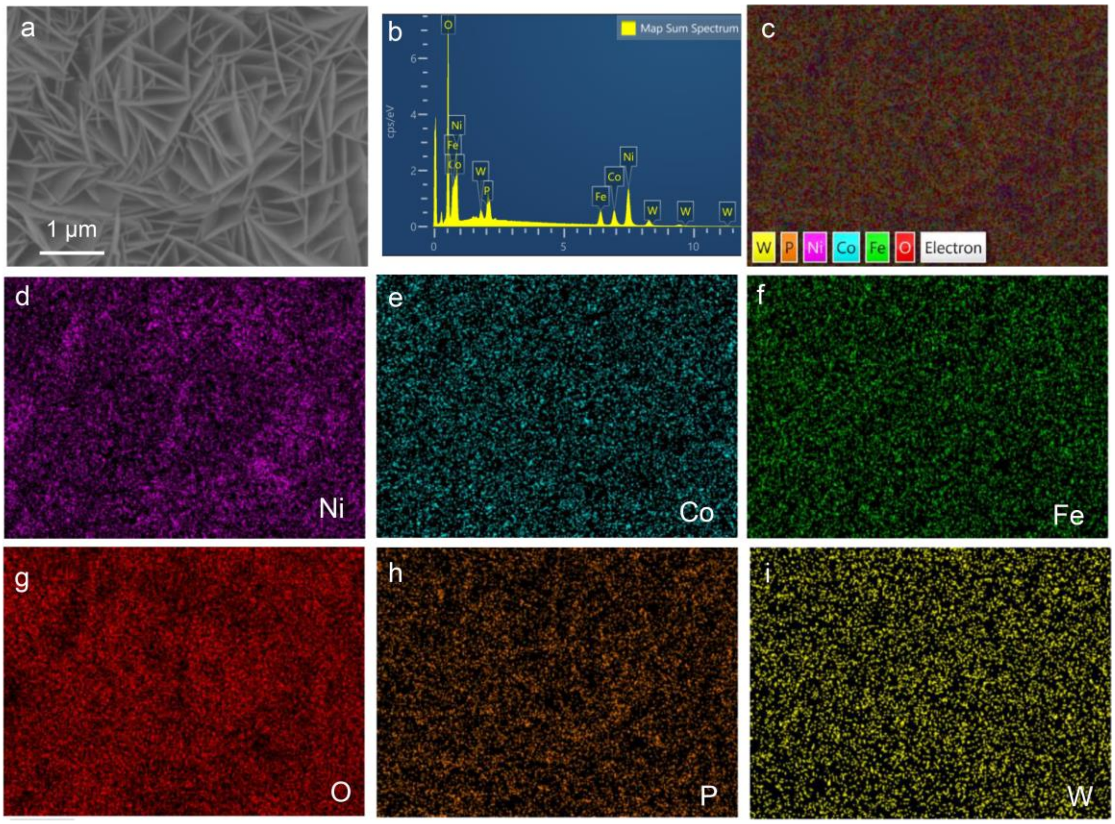

Supplementary Fig. 2 | Elemental distribution. (**a**) SEM image of PW_{12}-CoFe LDH/NF. (**b**) Energy-dispersive X-ray spectrum of PW_{12}-CoFe LDH/NF. (**c-i**) SEM image and its corresponding elemental mapping images of PW_{12}-CoFe LDH/NF.

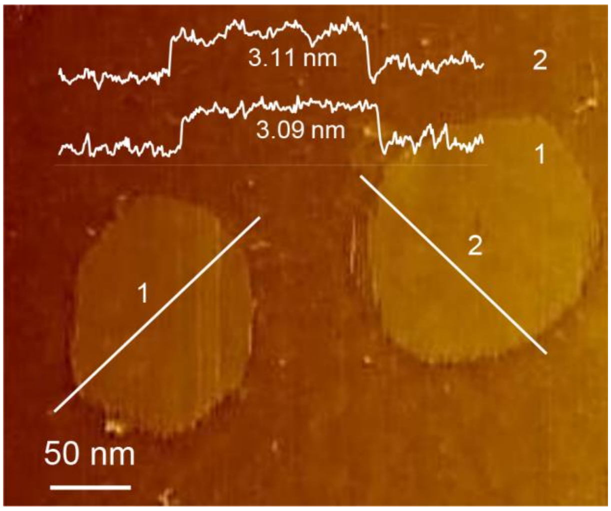

Supplementary Fig. 3 | Atomic force microscopy (AFM) image. AFM image of PW_{12}-CoFe LDH. Source data are provided as a Source Data file.

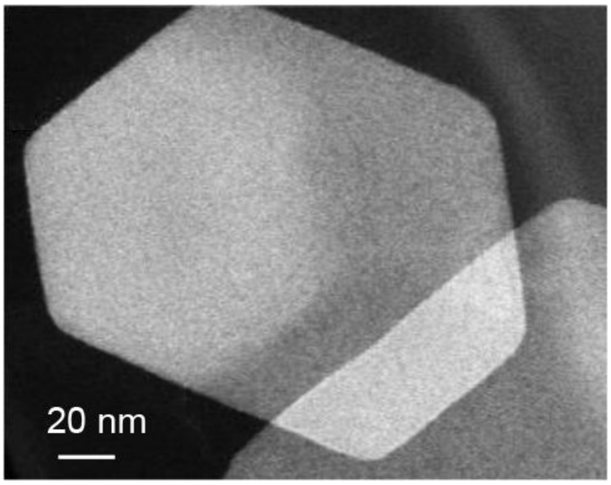

Supplementary Fig. 4 | AC-STEM image. AC-STEM image of PW_{12}-CoFe LDH.

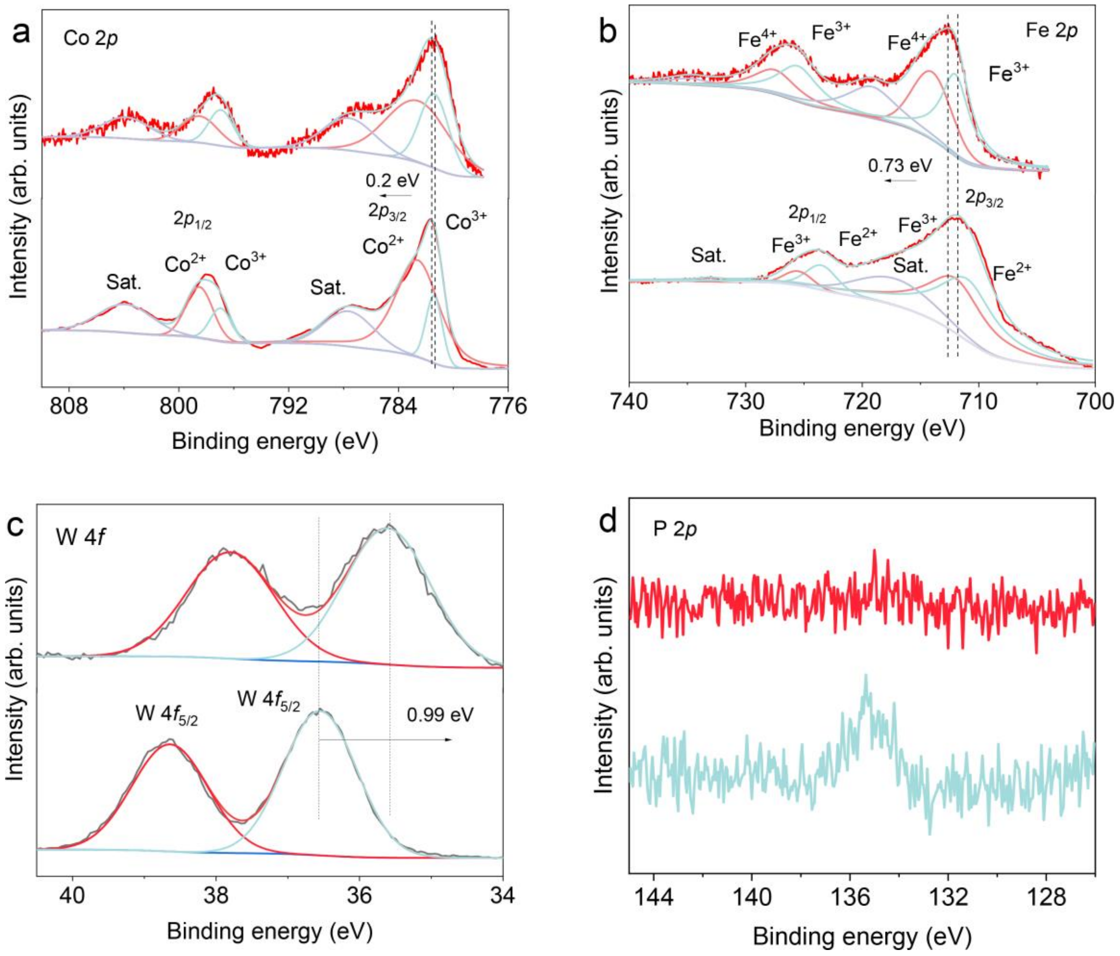

Supplementary Fig. 5 | XPS spectra. Comparison of XPS spectra for PW_{12}-CoFe LDH/NF (top) and CoFe LDH/NF (bottom) in the (**a**) Co 2*p* and (**b**) Fe 2*p* regions. Comparison of XPS spectra for PW_{12}-CoFe LDH/NF (top) and PW_{12}-POM (bottom) in the (**c**) W 4*f* and (**d**) P 2*p* regions. Source data are provided as a Source Data file.

Due to the low concentration of P content (0.36 wt%, Supplementary Table 1) in PW_{12}-CoFe LDH and a low P/W ratio for PW_{12}-CoFe LDH, the corresponding XPS signal intensity is weak, thus subsequent XANES spectrum and post-reaction XPS analyses of P could not be conducted reliably.

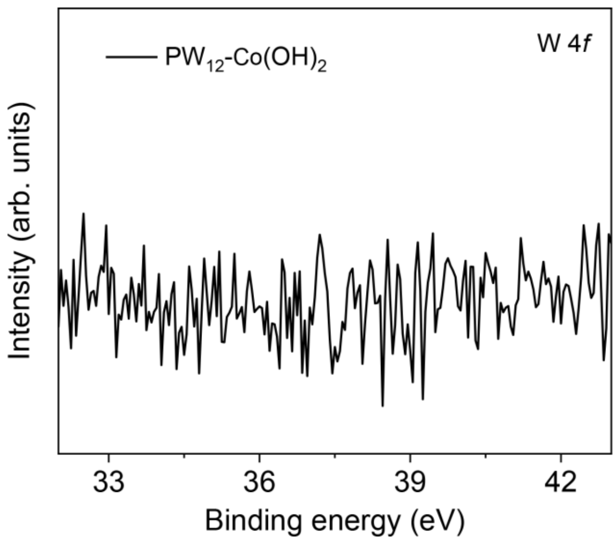

Supplementary Fig. 6 | XPS spectrum. XPS spectrum of PW_{12}-$Co(OH)_2$ in the W 4*f* region. Source data are provided as a Source Data file.

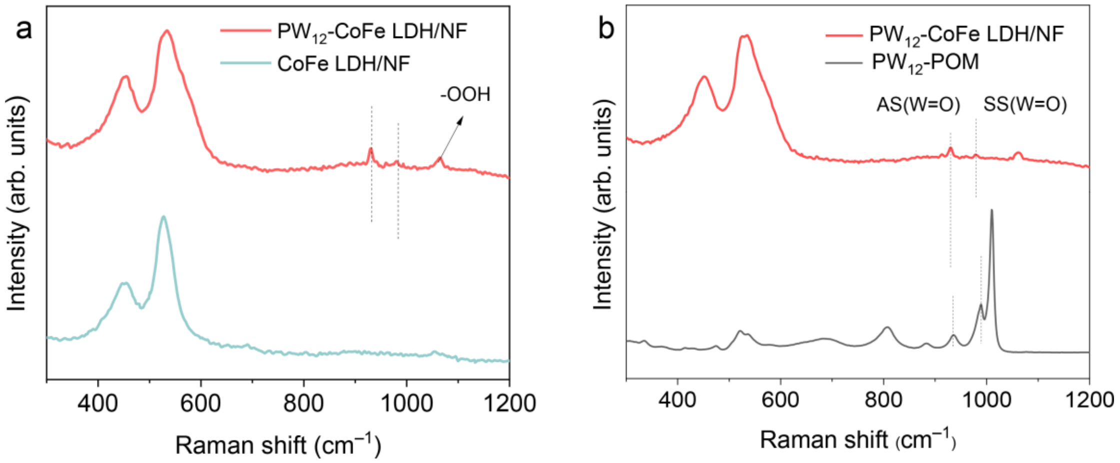

Supplementary Fig. 7 | Raman spectra. (**a**) Comparison of Raman spectra for CoFe LDH and PW_{12}-CoFe LDH. (**b**) Comparison of Raman spectra for PW_{12}-POM and PW_{12}-CoFe LDH. Source data are provided as a Source Data file.

The Raman peaks of CoFe LDH at 452.6 and 526.3 cm^{-1}, shift respectively to 452.8 and 529.4 cm^{-1} after PW_{12}-POM incorporation, with a notable increase in intensity at 452.8 cm^{-1} relative to 529.4 cm^{-1}, and the appearance of a new peak at 1060 cm^{-1}, assigned to the –OOH species (Supplementary Fig. 7a) (*Nano Energy* **83**, 105838 (2021); *Nano Today* **58**, 102454 (2024); *Nat. Commun.* **15**, 1973 (2024)). Additionally, the intensity of the terminal W=O asymmetric stretching vibration (AS(W=O)) relative to the terminal W=O symmetric stretching vibration (SS(W=O)) for PW_{12}-CoFe LDH is markedly enhanced (*Catal. Lett.* **153**, 3092–3102 (2023); *ACS Catal.* **8**, 2330–2342 (2018); *Environ. Eng. Sci.* **32**, 3, (2015)). These observations imply that PW_{12}-POM form strong chemical coordination with CoFe LDH and promote the dehydrogenation.

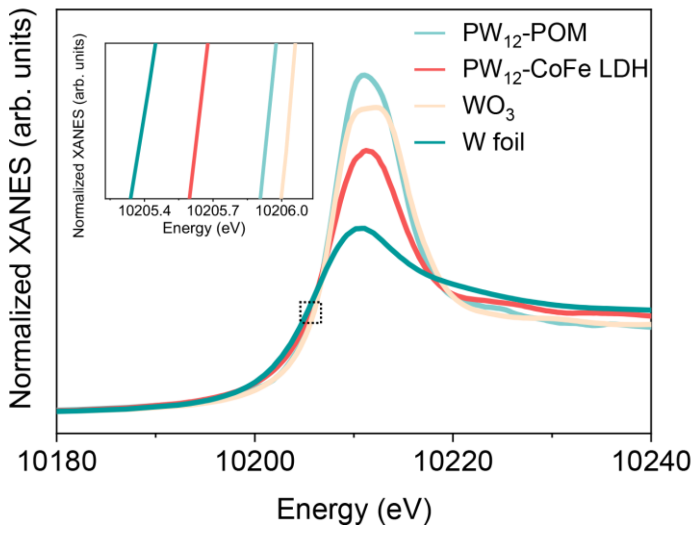

Supplementary Fig. 8 | XANES spectra. Normalized W L_3-edge XANES spectra of PW_{12}-CoFe LDH, CoFe LDH, WO_3, and W foil. Source data are provided as a Source Data file.

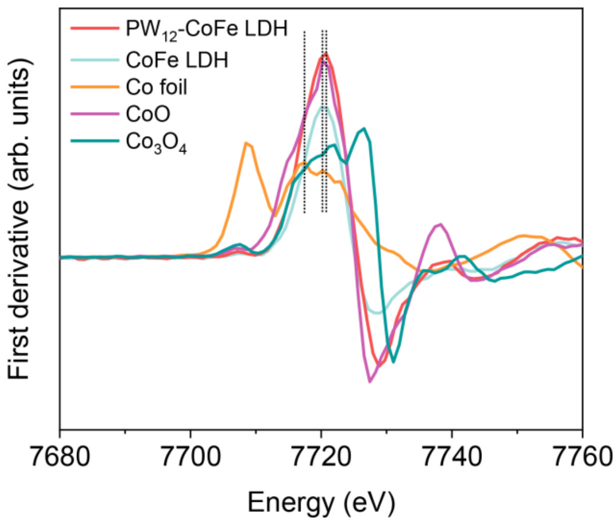

Supplementary Fig. 9 | First derivative spectra. First derivative spectra for the experimental Co K-edge of CoFe LDH, PW_{12}-CoFe LDH, Co foil, CoO, and Co_3O_4. Source data are provided as a Source Data file.

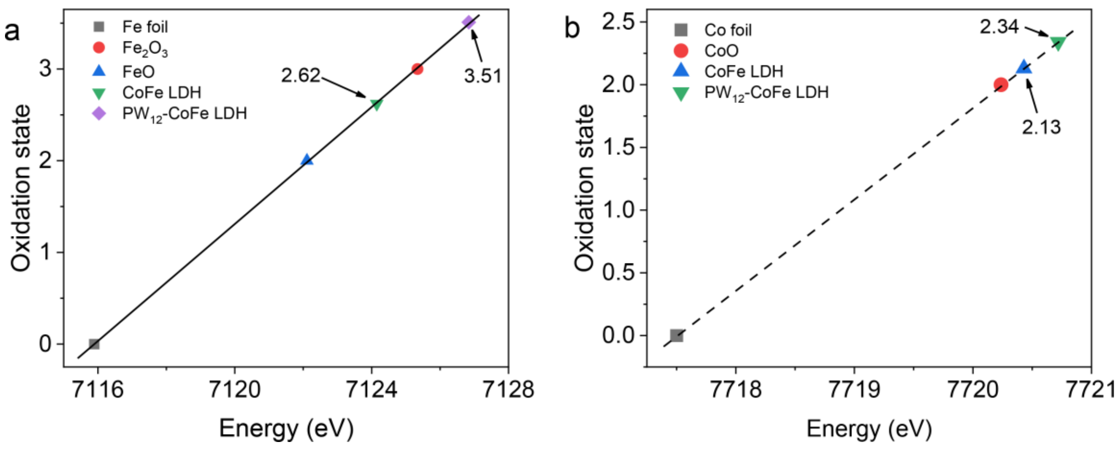

Supplementary Fig. 10 | Oxidation states for Fe and Co. (**a**) Oxidation states of Fe for CoFe LDH, PW_{12}-CoFe LDH, Fe_2O_3, FeO, and Fe foil. (**b**) Oxidation states of Co for CoFe LDH, PW_{12}-CoFe LDH, Co foil, and CoO.

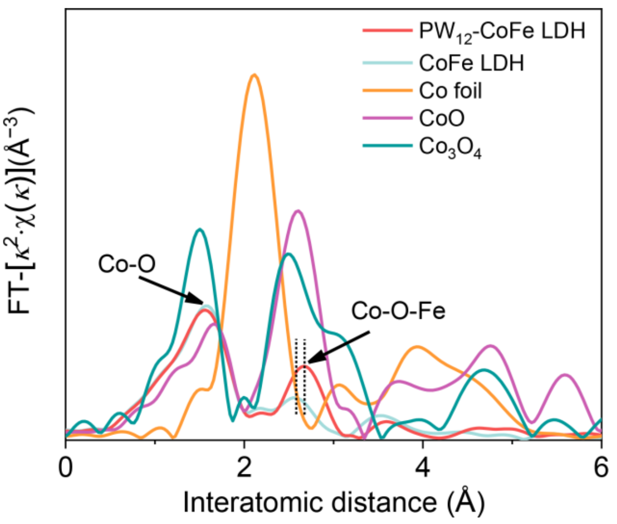

Supplementary Fig. 11 | FT-EXAFS spectra. FT-EXAFS spectra of CoFe LDH, PW_{12}-CoFe LDH, Co foil, CoO, and Co_3O_4. Source data are provided as a Source Data file.

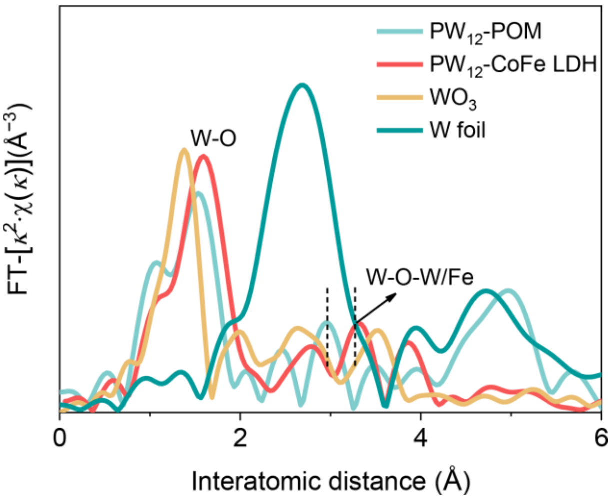

Supplementary Fig. 12 | FT-EXAFS spectra. FT-EXAFS spectra of PW_{12}-CoFe LDH, PW_{12}-POM, WO_3, and W foil. Source data are provided as a Source Data file.

The increase for the coordination number and elongation of W–O bonds, along with the stretched W–O–W interactions, further indicate strong interfacial coordination between PW_{12}-POM and CoFe LDH.

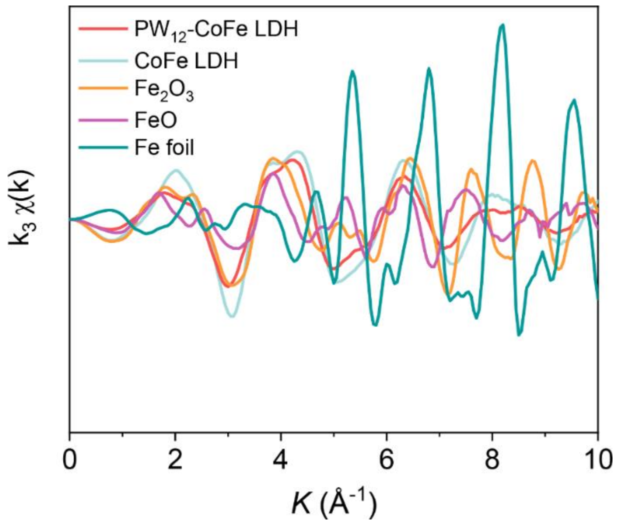

Supplementary Fig. 13 | Fe K-edge EXAFS. Fe K-edge EXAFS oscillation function of CoFe LDH, PW_{12}-CoFe LDH, Fe_2O_3, FeO, and Fe foil. Source data are provided as a Source Data file.

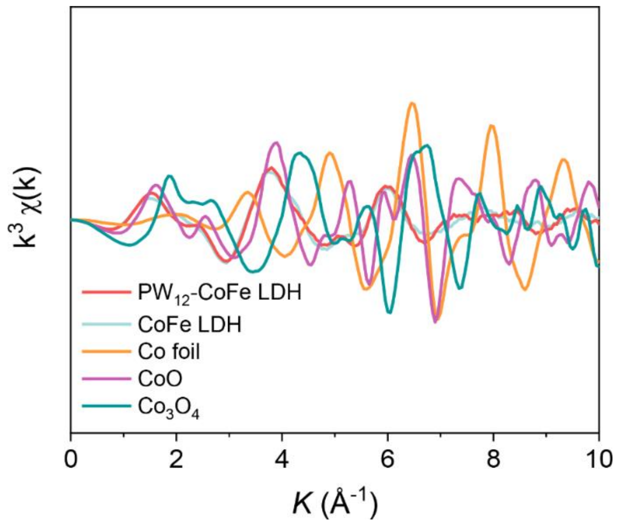

Supplementary Fig. 14 | Co K-edge EXAFS. Co K-edge EXAFS oscillation function of CoFe LDH, PW_{12}-CoFe LDH, Co foil, CoO, and Co_3O_4. Source data are provided as a Source Data file.

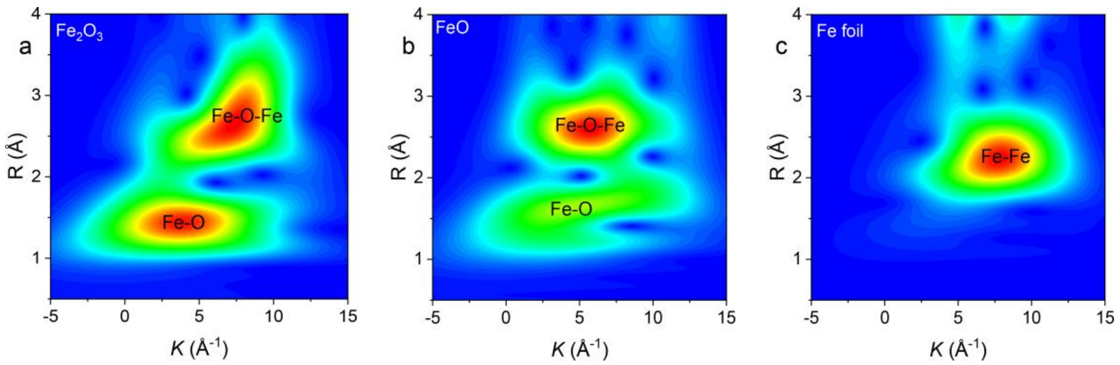

Supplementary Fig. 15 | Wavelet transformed EXAFS. Wavelet transformed EXAFS plots of (**a**) Fe_2O_3, (**b**) FeO, and (**c**) Fe foil.

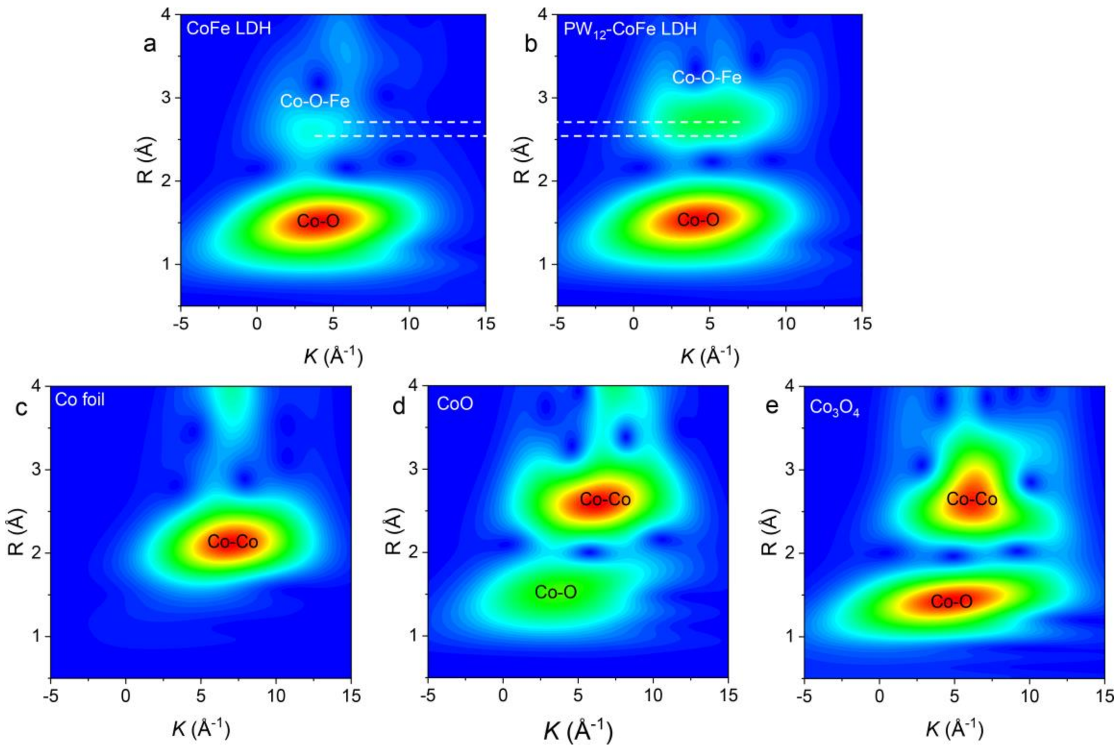

Supplementary Fig. 16 | Wavelet transformed EXAFS. Wavelet transformed EXAFS plots of (**a**) CoFe LDH, (**b**) PW_{12}-CoFe LDH, (**c**) Co foil, (**d**) CoO, and (**e**) Co_3O_4.

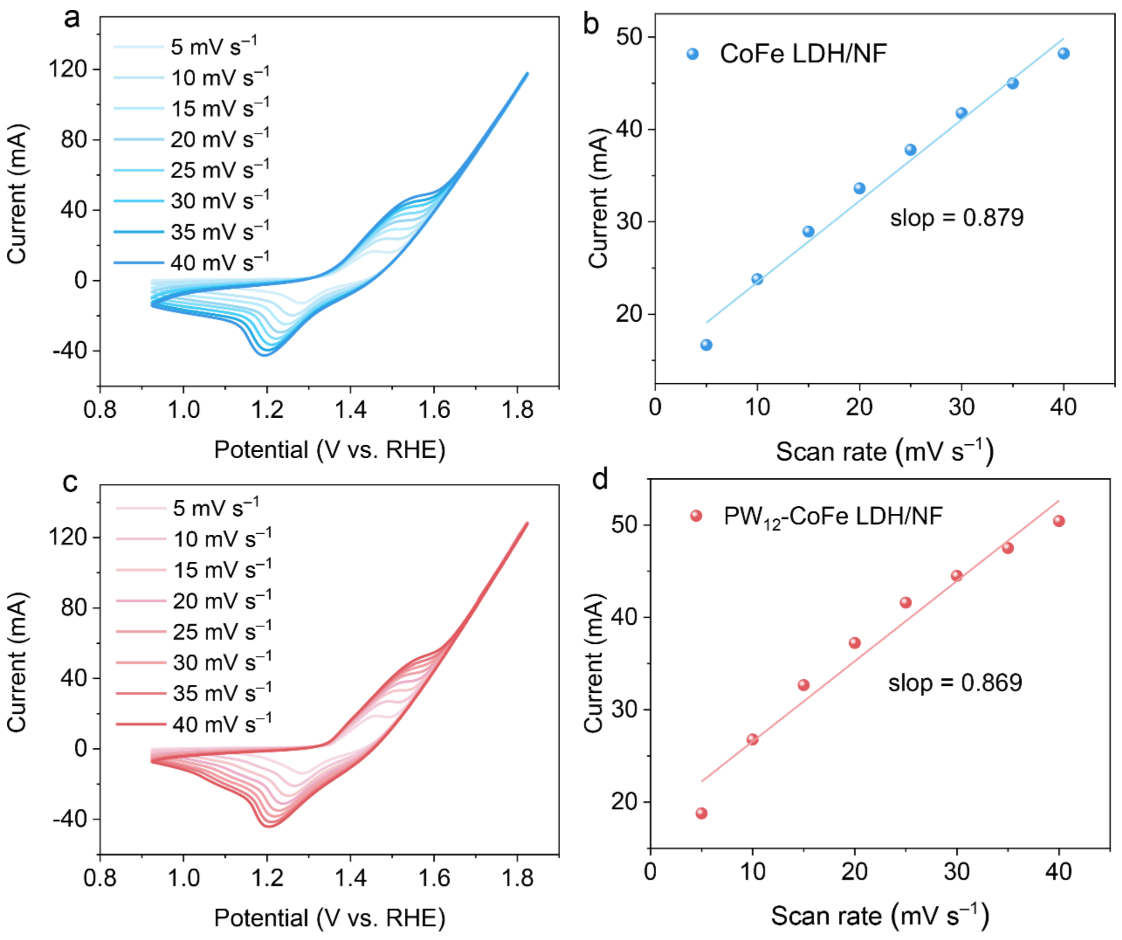

Supplementary Fig. 17 | Cyclic voltammetry (CV) curves and relationships between oxidation peak currents and scan rates. CV curves of (**a**) CoFe LDH/NF and (**c**) PW_{12}-CoFe LDH/NF, and its corresponding relationships between oxidation peak currents and scan rates of (**b**) CoFe LDH/NF and (**d**) PW_{12}-CoFe LDH/NF, respectively. Slopes of 0.879 ± 0.02 for CoFe LDH/NF and 0.869 ± 0.04 for PW_{12}-CoFe LDH/NF were obtained from triplicate measurements; only representative data are shown. Source data are provided as a Source Data file.

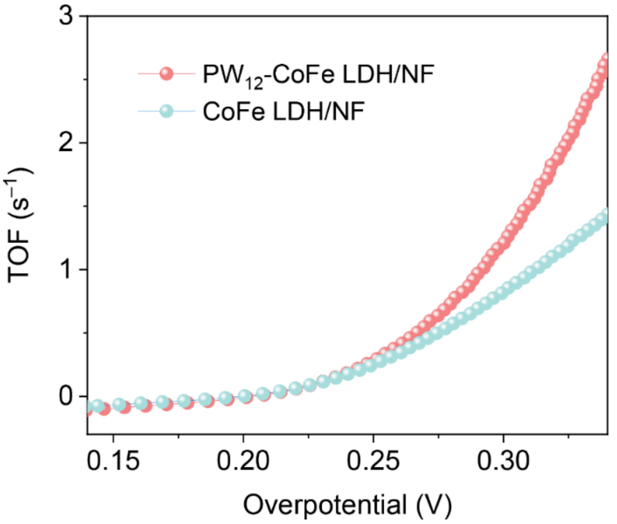

Supplementary Fig. 18 | Turnover frequency (TOF). TOF values of the PW_{12}-CoFe LDH/NF and CoFe LDH/NF electrodes. Source data are provided as a Source Data file.

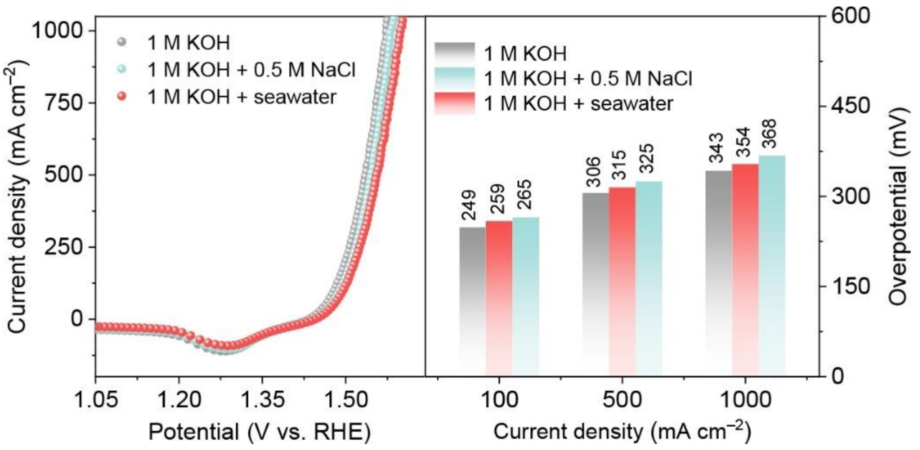

Supplementary Fig. 19 | Evaluation of activities. Polarization curves (left) and comparison of overpotentials (right) for the PW_{12}-CoFe LDH/NF anode in 1 M KOH, 1 M KOH + 0.5 M NaCl, and 1 M KOH + seawater. Source data are provided as a Source Data file.

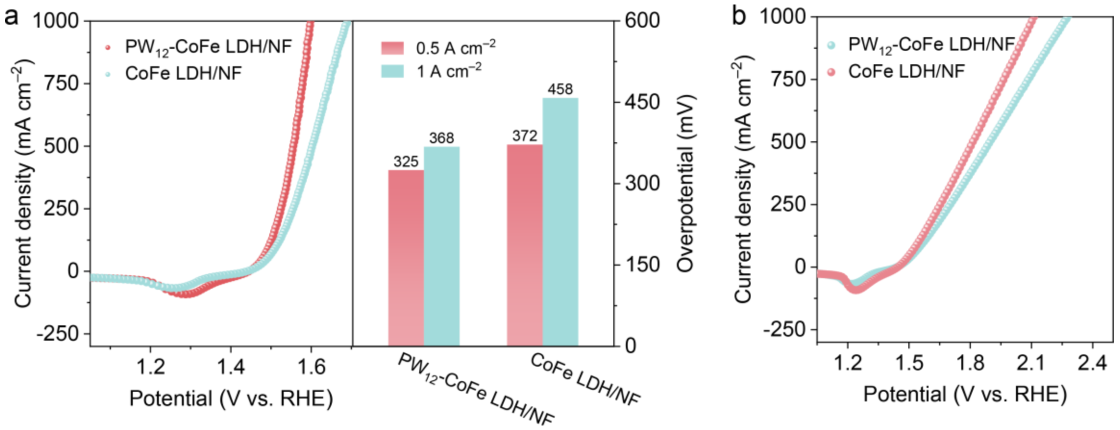

Supplementary Fig. 20 | Evaluation of activities. (**a**) Polarization curves (left) and comparison of overpotentials (right) for the PW_{12}-CoFe LDH/NF and CoFe LDH/NF electrodes in 1 M KOH + seawater with 100% *iR* compensation. (**b**) Polarization curves of the PW_{12}-CoFe LDH/NF and CoFe LDH/NF electrodes in 1 M KOH + seawater without *iR* compensation. The corresponding solution resistances are 2.055 ± 0.04 Ω for PW_{12}-CoFe LDH/NF and 2.349± 0.06 Ω for CoFe LDH/NF. The geometric area of all electrodes is 0.25 cm^2. Source data are provided as a Source Data file.

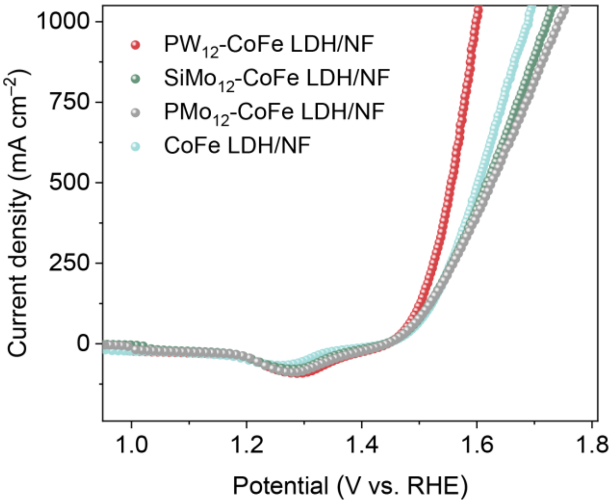

Supplementary Fig. 21 | Evaluation of activities. Comparison of polarization curves for CoFe LDH/NF, SiW_{12}-CoFe LDH/NF, PMo_{12}-CoFe LDH/NF, and PW_{12}-CoFe LDH/NF with 100% *iR* compensation. Source data are provided as a Source Data file.

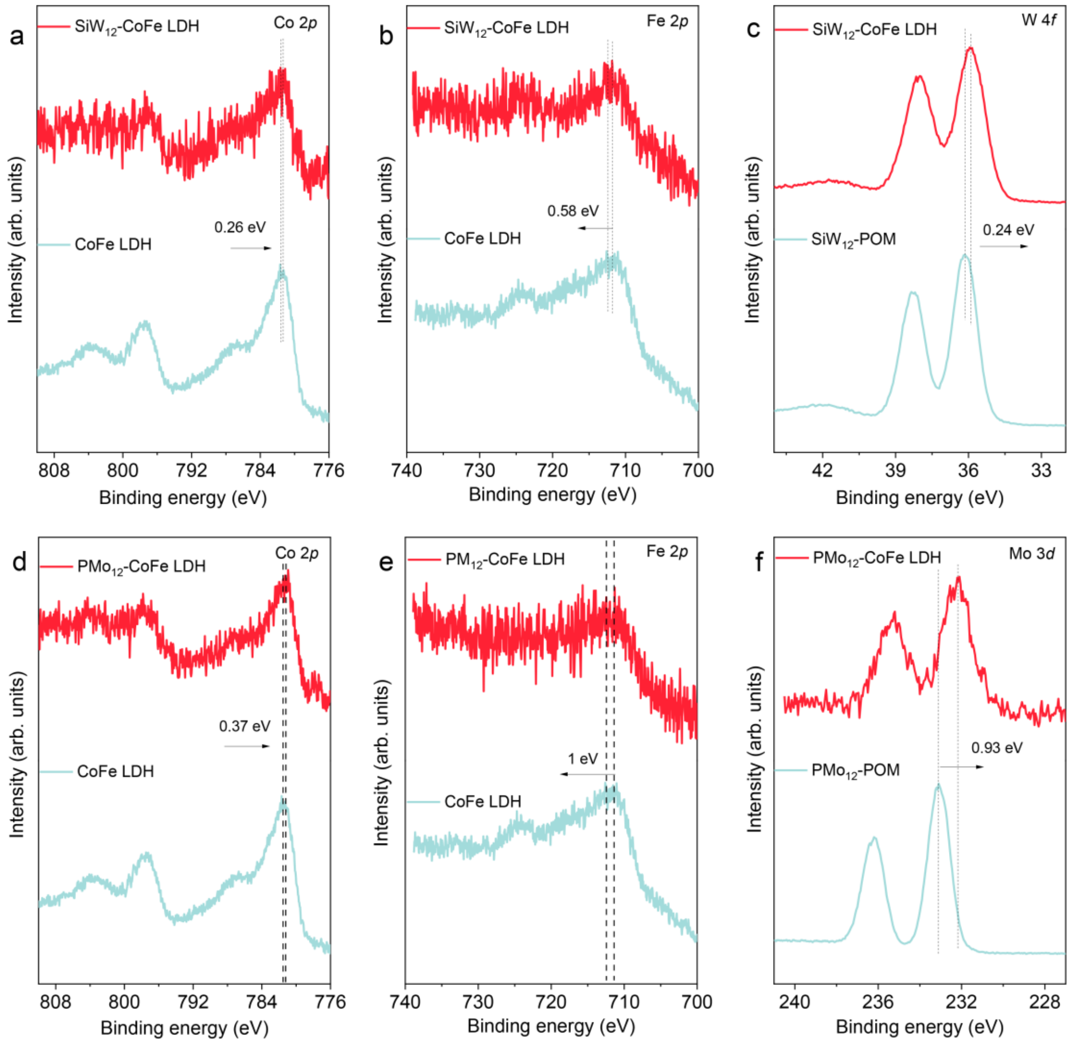

Supplementary Fig. 22 | XPS spectra. Comparison of XPS spectra for SiW_{12}-CoFe LDH/NF and CoFe LDH/NF in the (**a**) Co 2*p* and (**b**) Fe 2*p* regions. (**c**) Comparison of XPS spectra for SiW_{12}-CoFe LDH/NF and SiW_{12}-POM in the W 4*f* region. Comparison of XPS spectra for PMo_{12}-CoFe LDH/NF and CoFe LDH/NF in the (**d**) Co 2*p* and (**e**) Fe 2*p* regions. (**f**) Comparison of XPS spectra for PMo_{12}-CoFe LDH/NF and PMo_{12}-POM in the Mo 3*d* region. Source data are provided as a Source Data file.

For SiW_{12}-CoFe LDH/NF and PMo_{12}-CoFe LDH/NF, the Fe 2*p* peaks shift positively to higher binding energies and the W 4*f* and Mo 3*d* peaks shift negatively to lower binding energies (Supplementary Fig. 22), matching the trend observed with PW_{12}-CoFe LDH/NF. In contrast, Co 2*p* peaks exhibit negative shifts, indicating that Co atoms could gain electrons and lower their oxidation state, thereby potentially reducing catalytic efficiency.

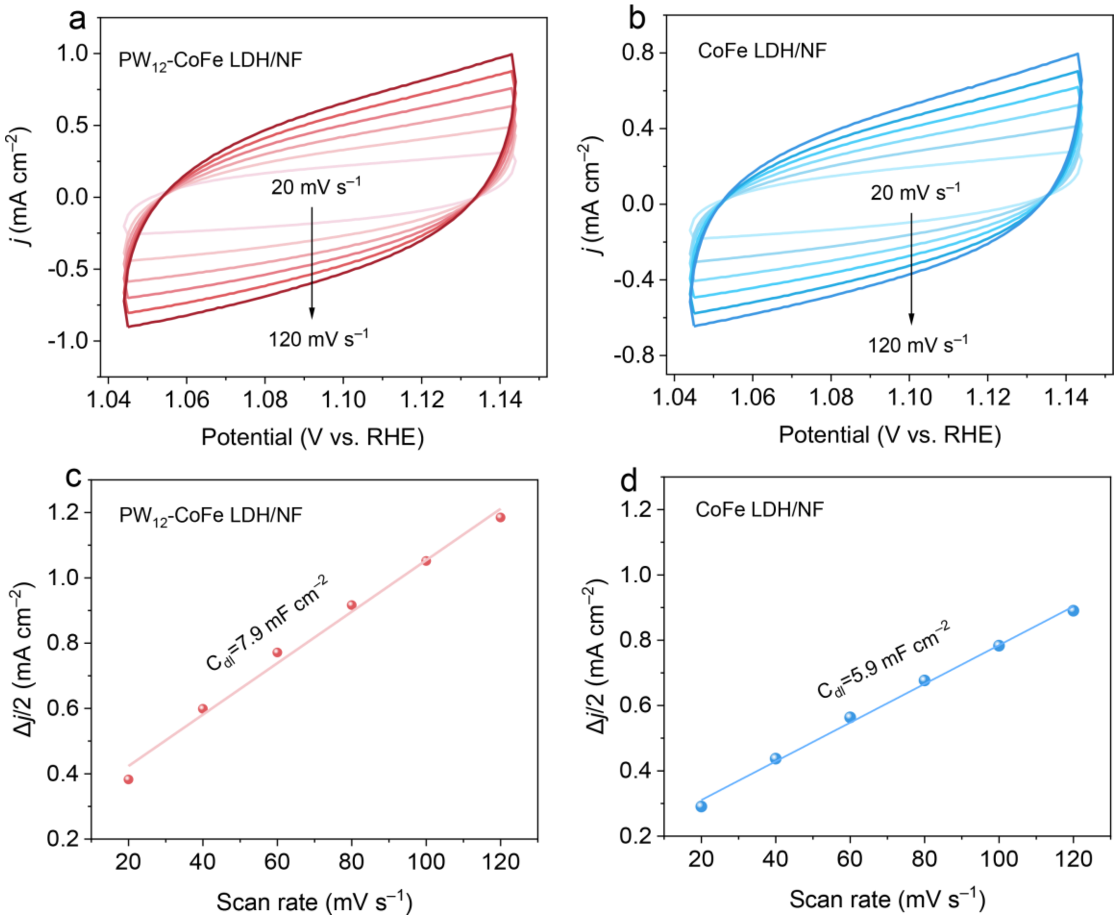

Supplementary Fig. 23 | Electrochemical double-layer capacities (C_{dl}) measurements. CV curves in the non-Faradaic zone at various scan rates for (**a**) PW_{12}-CoFe LDH/NF and (**b**) CoFe LDH/NF electrodes in 1 M KOH + seawater without *iR* correction. The C_{dl} values for (**c**) PW_{12}-CoFe LDH/NF and (**d**) CoFe LDH/NF electrodes. Source data are provided as a Source Data file.

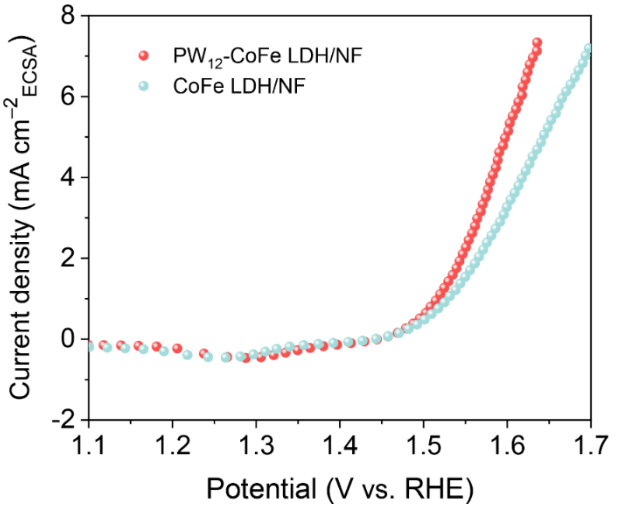

Supplementary Fig. 24 | Evaluation of activities. Polarization curves for PW_{12}-CoFe LDH/NF and CoFe LDH/NF for ASO with ECSA fitting with 100% *iR* correction. Source data are provided as a Source Data file.

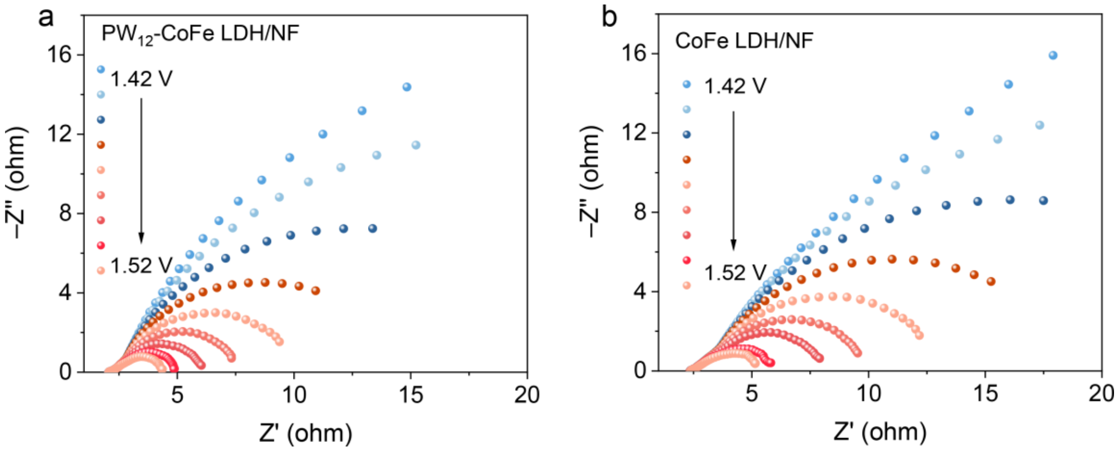

Supplementary Fig. 25 | Evaluation of kinetics. Electrochemical impedance spectroscopy (EIS) Nyquist plots collected from 1.42-1.52 V over a frequency range of 10^{-2} to 10^{5} Hz for (**a**) PW_{12}-CoFe LDH/NF and (**b**) CoFe LDH/NF electrodes during ASO. Source data are provided as a Source Data file.

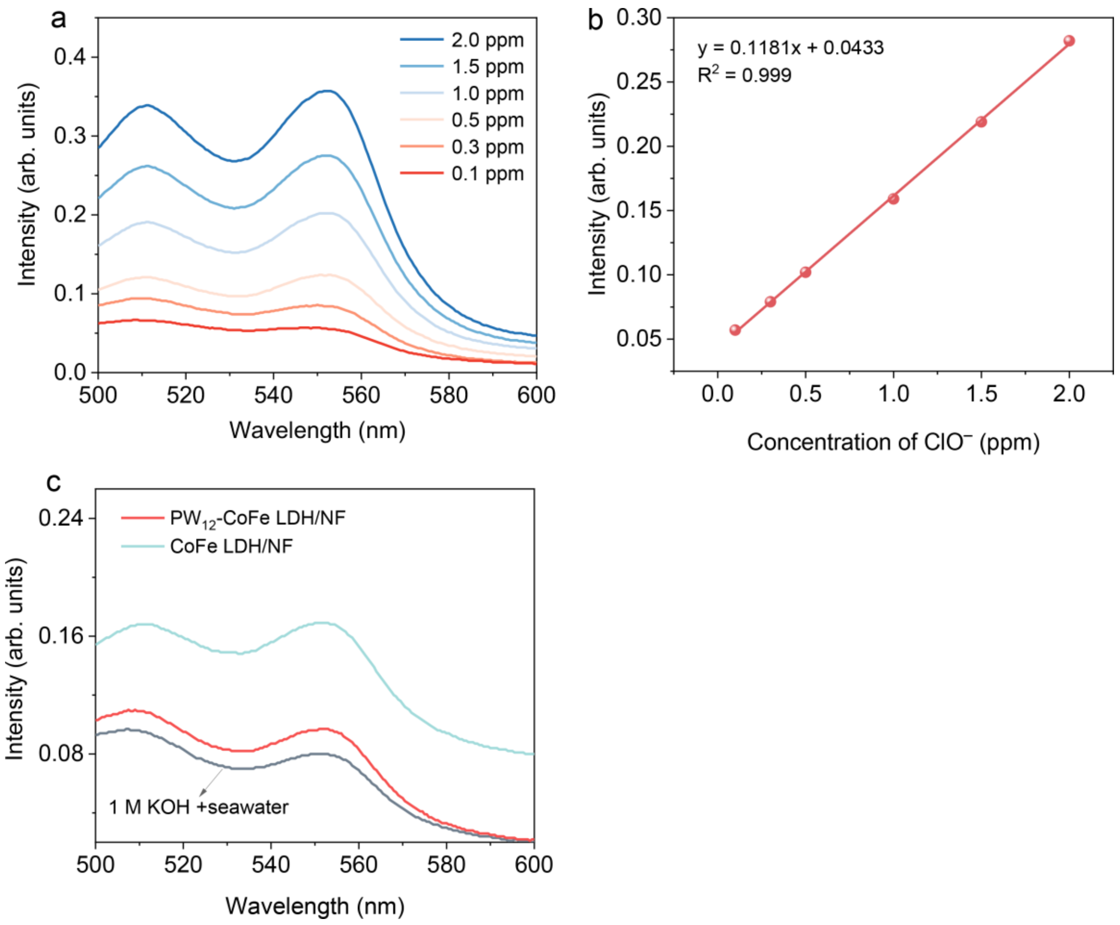

Supplementary Fig. 26 | Chlorine precipitation tests. (**a**) Ultraviolet–visible (UV–vis) absorption spectra of various concentrations of active chlorine. (**b**) Calibration curve. (**c**) UV-vis absorption spectra of the blank electrolyte, PW_{12}-CoFe LDH/NF electrode electrolyte, and CoFe LDH/NF electrode electrolyte after stability tests at 1 A cm^{-2}. Source data are provided as a Source Data file.

The active chlorine concentration in the electrolyte was determined using UV-vis spectroscopy via the DPD colorimetric method (*J. Electroanal. Chem*. **819**, 260–268 (2018); *Nano Today* **58**, 102454 (2024)). After stability tests at 1 A cm^{-2}, 100 μL electrolyte was mixed with 50 μL of 1.0 M H_2SO_4 and 2.0 M NaOH, plus 4.8 mL of deionized water. The sample, mixed with 250 μL of DPD reagent and phosphate-buffered saline (pH 6.5), shows a pink color, and chlorine is detected spectrophotometrically at 550 nm.

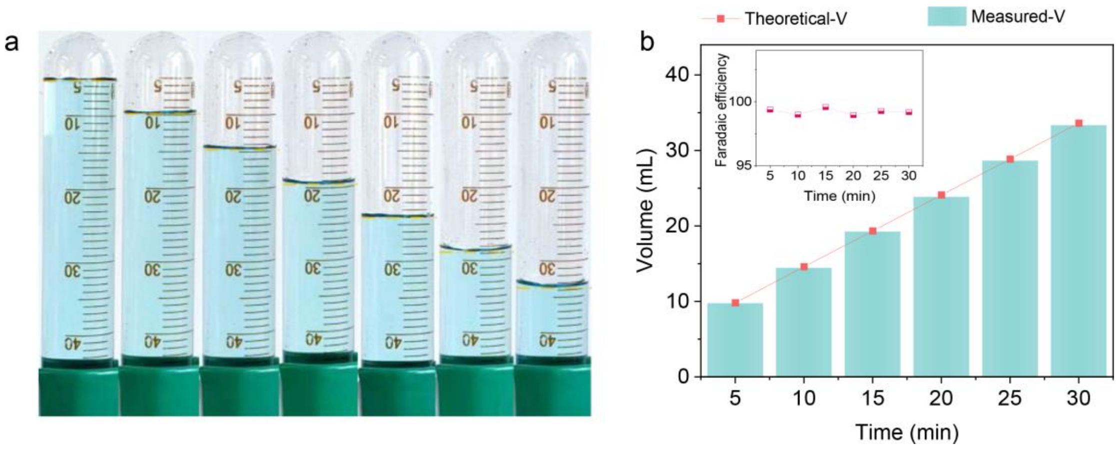

Supplementary Fig. 27 | O_2 Faradaic efficiency measurements. (**a**) Digital photographs of the collected O_2 during ASO. (**b**) Comparison of collected O_2 with theoretical values for the PW_{12}-CoFe LDH/NF electrode at 1 A cm^{-2} in 1 M KOH + seawater. Source data are provided as a Source Data file.

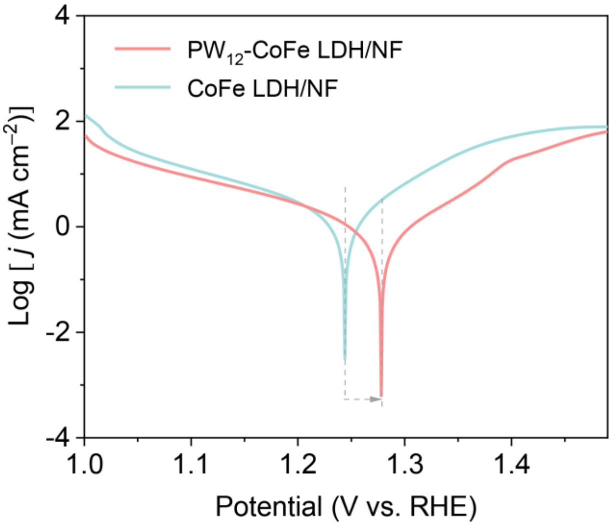

Supplementary Fig. 28 | Corrosion behavior curves. Comparison of the corrosion behavior curves for the CoFe LDH/NF and PW_{12}-CoFe LDH/NF anodes. Source data are provided as a Source Data file.

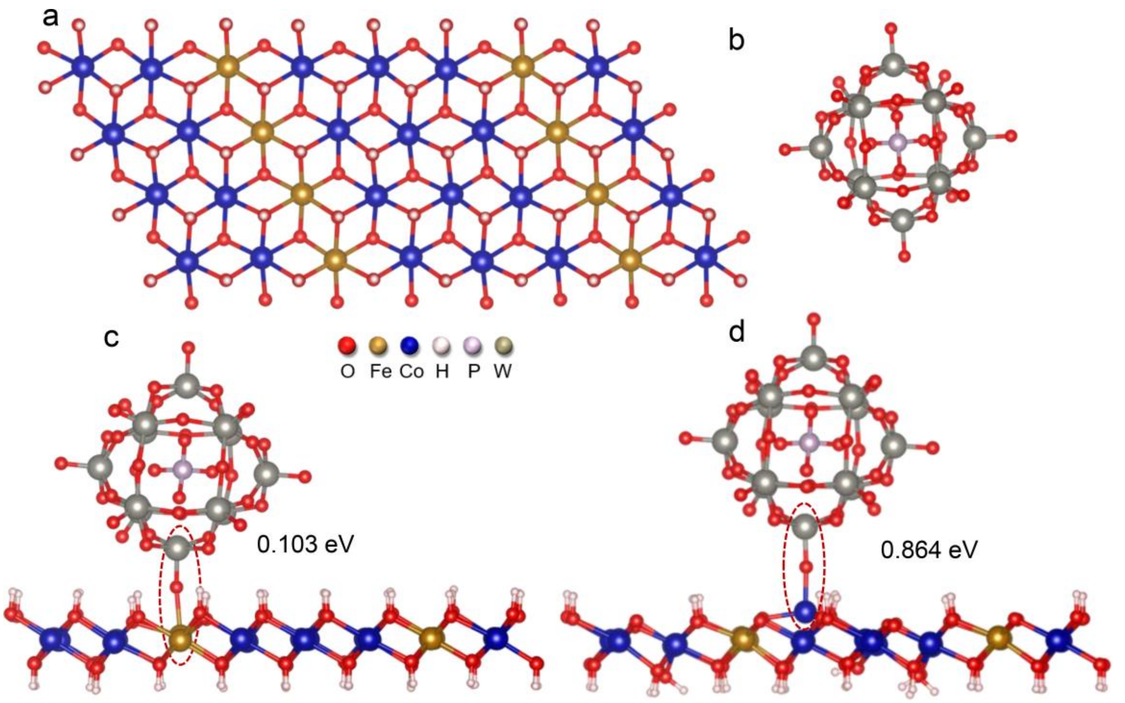

Supplementary Fig. 29 | Structural diagrams. Structural diagrams of (**a**) CoFe LDH. (**b**) PW_{12}-POM. Structural diagrams of PW_{12}-CoFe LDH connected via (**c**) Fe and (**d**) Co sites.

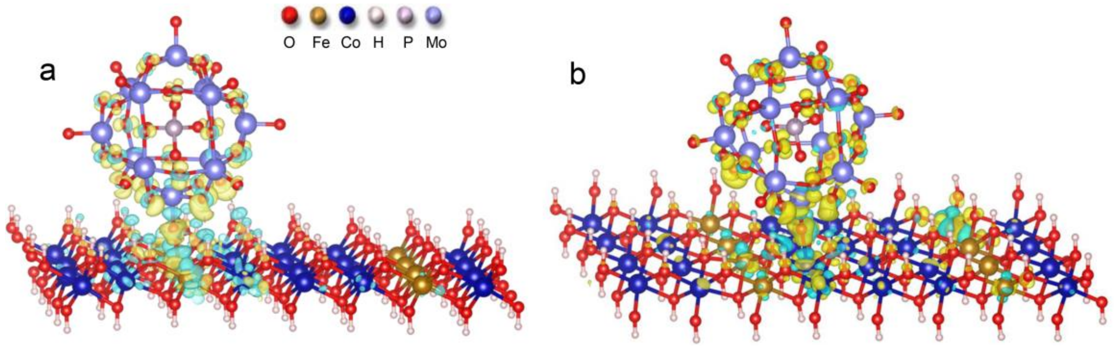

Supplementary Fig. 30 | Charge density difference diagram of PMo_{12}-CoFe LDH connected via (a) Fe and (b) Co sites. Yellow: charge accumulation; cyan: charge depletion.

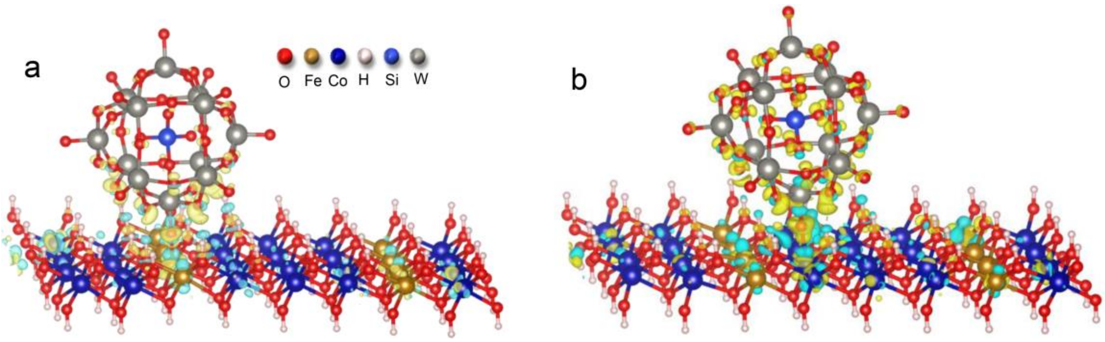

Supplementary Fig. 31 | Charge density difference diagram of SiW_{12}-CoFe LDH connected via (a) Fe and (b) Co sites.

In comparison, SiW_{12}-POM and PMo_{12}-POM display smaller energy differences between Fe and Co adsorption on CoFe LDH (SiW_{12}-POM: Fe 0.461 eV, Co 0.776 eV; PMo_{12}-POM: Fe 0.380 eV, Co 0.816 eV) (Supplementary Fig. 30, 31), suggesting competitive adsorption of Fe and Co. Correspondingly, XPS spectra show positive shifts in Fe 2*p* binding energies along with negative shifts in W 4*f* and Mo 3*d*, indicating that electrons are primarily transferred from Fe to the SiW_{12}-POM and PMo_{12}-POM. Charge density difference diagrams further reveal that, while Fe sites serve as the major electron donors, a minor portion of electrons can accumulate toward Co when SiW_{12}-POM or PMo_{12}-POM are adsorbed at Co site. This localized back-transfer could explain the slight electron accumulation at Co and account for the modest negative shifts observed in the Co 2*p* binding energies, thereby reducing catalytic efficiency. The highly electronegative central P^{5+} and stable W^{6+} center in PW_{12}-POM may strongly withdraw electrons from metal sites. The weaker acidity of SiW_{12}-POM resulting from its Si^{4+} center and the readily reducible Mo^{6+} center in PMo_{12}-POM may restrict effective electron extraction, thereby impacting their catalytic activity.

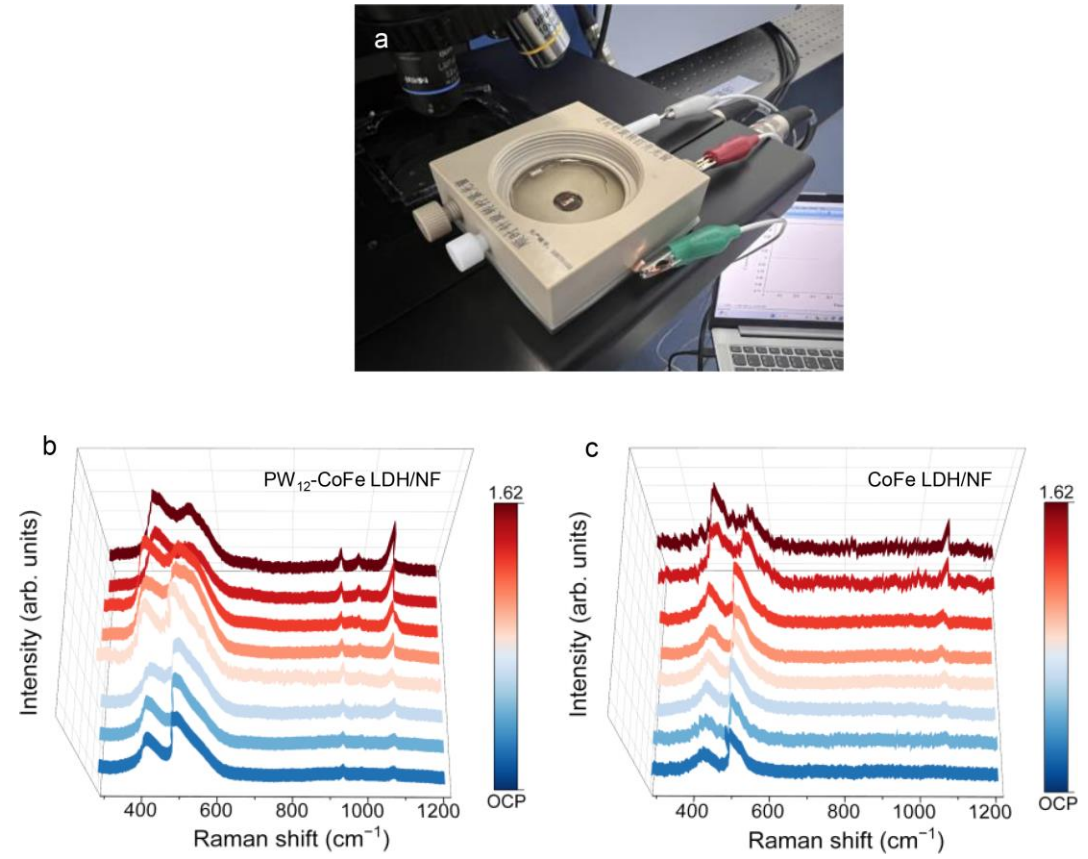

Supplementary Fig. 32 | Operando Raman spectra. (**a**) Photograph of the electrochemical cell used for in situ Raman measurements. Operando Raman spectra from OCP to 1.62 V *vs*. RHE for (**b**) PW_{12}-CoFe LDH/NF and (**c**) CoFe LDH/NF. Source data are provided as a Source Data file.

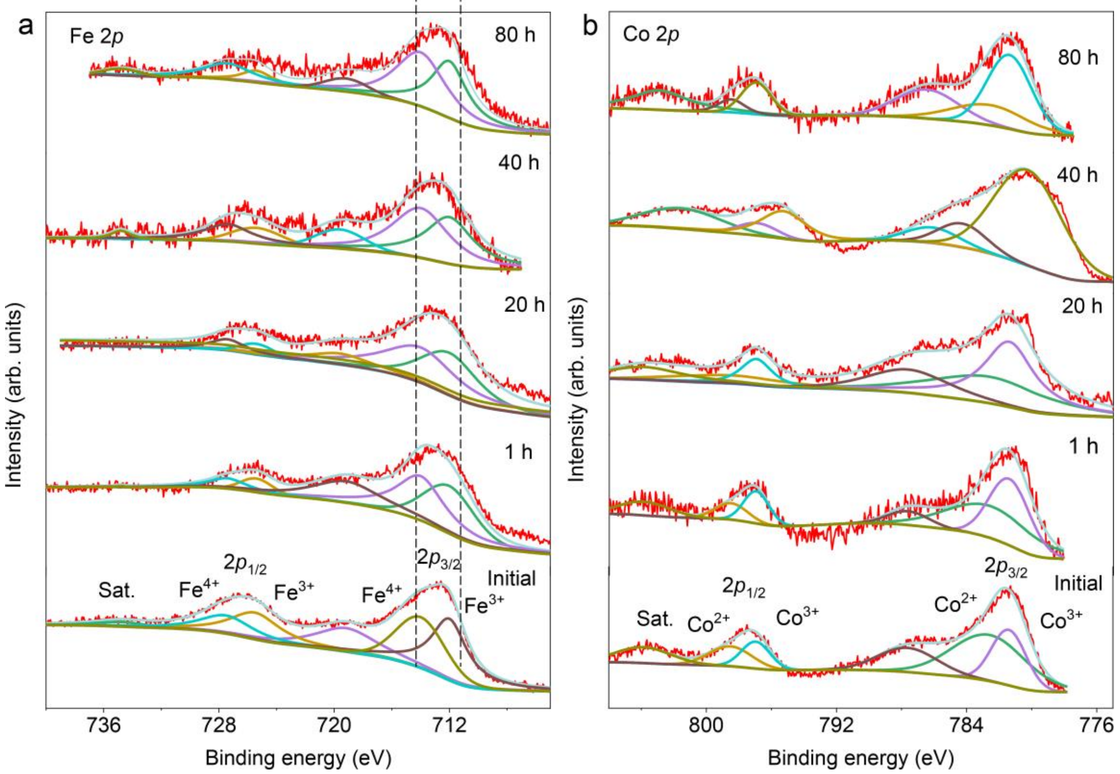

Supplementary Fig. 33 | Ex situ XPS tests. XPS spectra of PW_{12}-CoFe LDH/NF collected at different times in the (a) Fe 2*p* and (b) Co 2*p* regions. Source data are provided as a Source Data file.

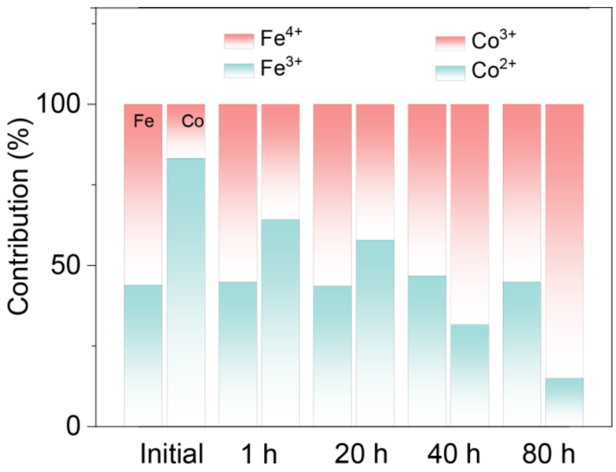

Supplementary Fig. 34 | Time-dependent valence state variations of Fe and Co species for POM-CoFe LDH/NF during ASO. Source data are provided as a Source Data file.

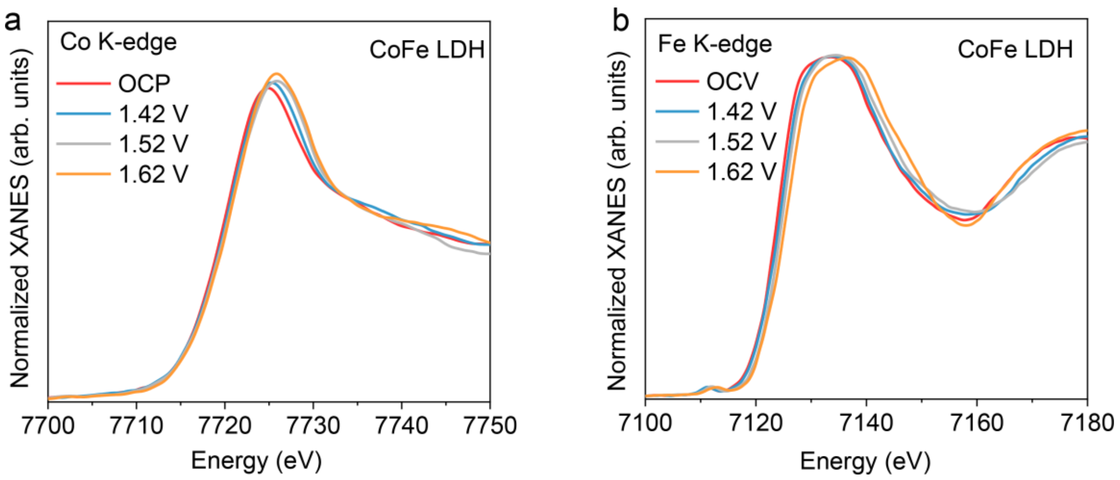

Supplementary Fig. 35 | In-situ XAS tests during ASO. In-situ (**a**) Co and (**b**) Fe K-edge XANES spectra of PW_{12}-CoFe LDH. Source data are provided as a Source Data file.

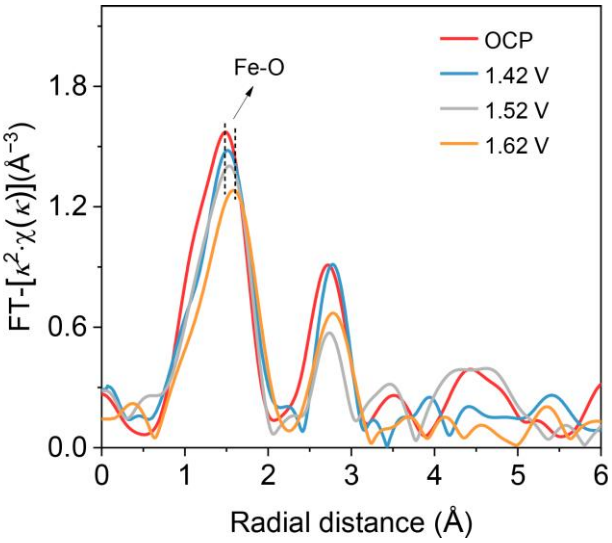

Supplementary Fig. 36 | In-situ XAS tests during ASO. In situ FT-EXAFS of Fe K-edge spectra for CoFe LDH. Source data are provided as a Source Data file.

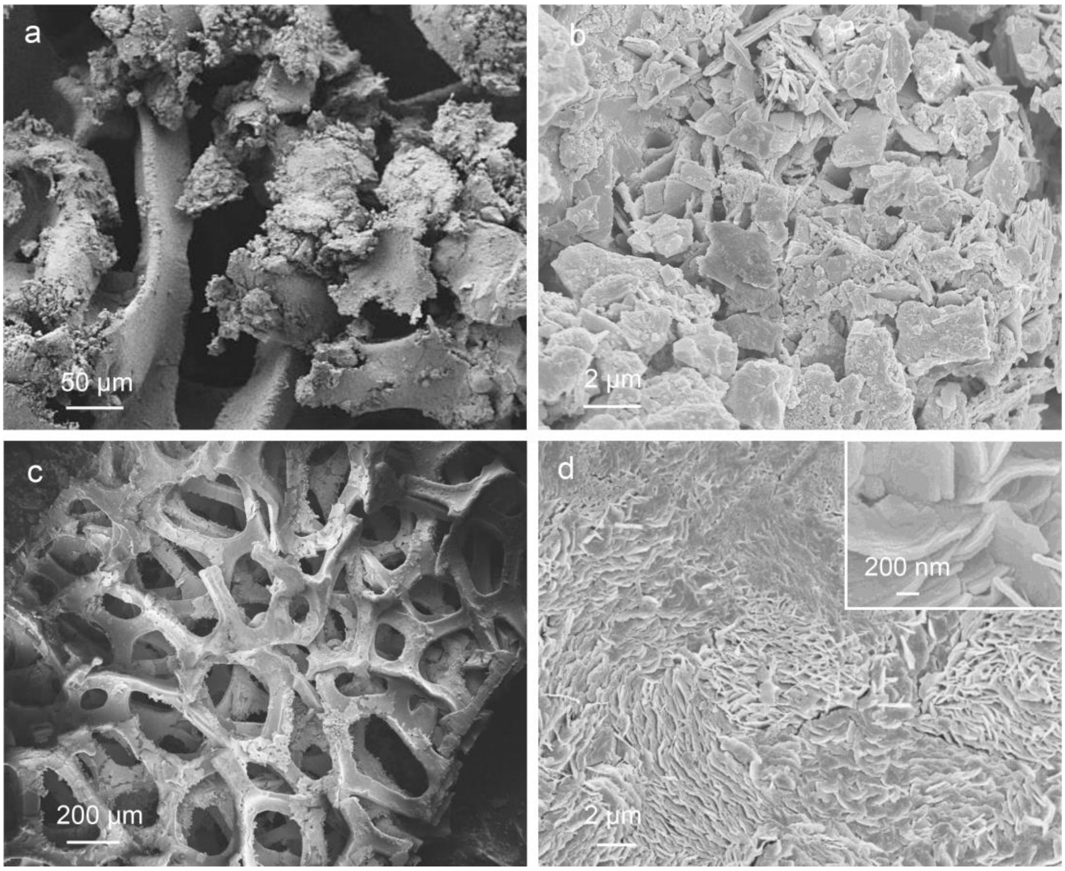

Supplementary Fig. 37 | SEM characterizations after stability tests. (**a**) Low-magnification and (**b**) high-magnification SEM images of CoFe LDH/NF after 80 hours of electrolysis. (**c**) Low-magnification and (**d**) high-magnification SEM images of PW_{12}-CoFe LDH/NF after 1300 hours of electrolysis.

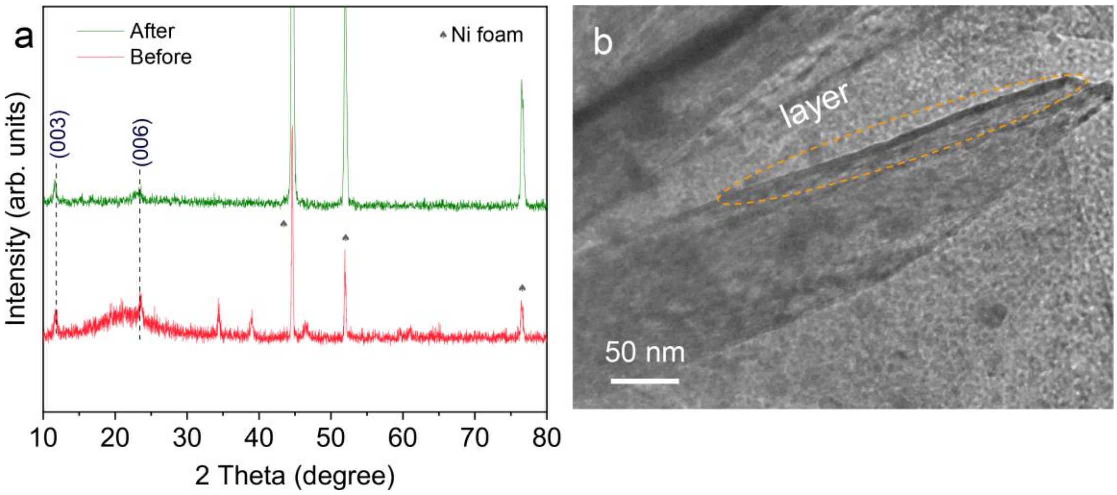

Supplementary Fig. 38 | XRD and TEM characterizations after stability tests. (**a**) XRD pattern of PW_{12}-CoFe LDH/NF and (**b**) TEM image of PW_{12}-CoFe LDH after 1300 hours of electrolysis. Source data are provided as a Source Data file.

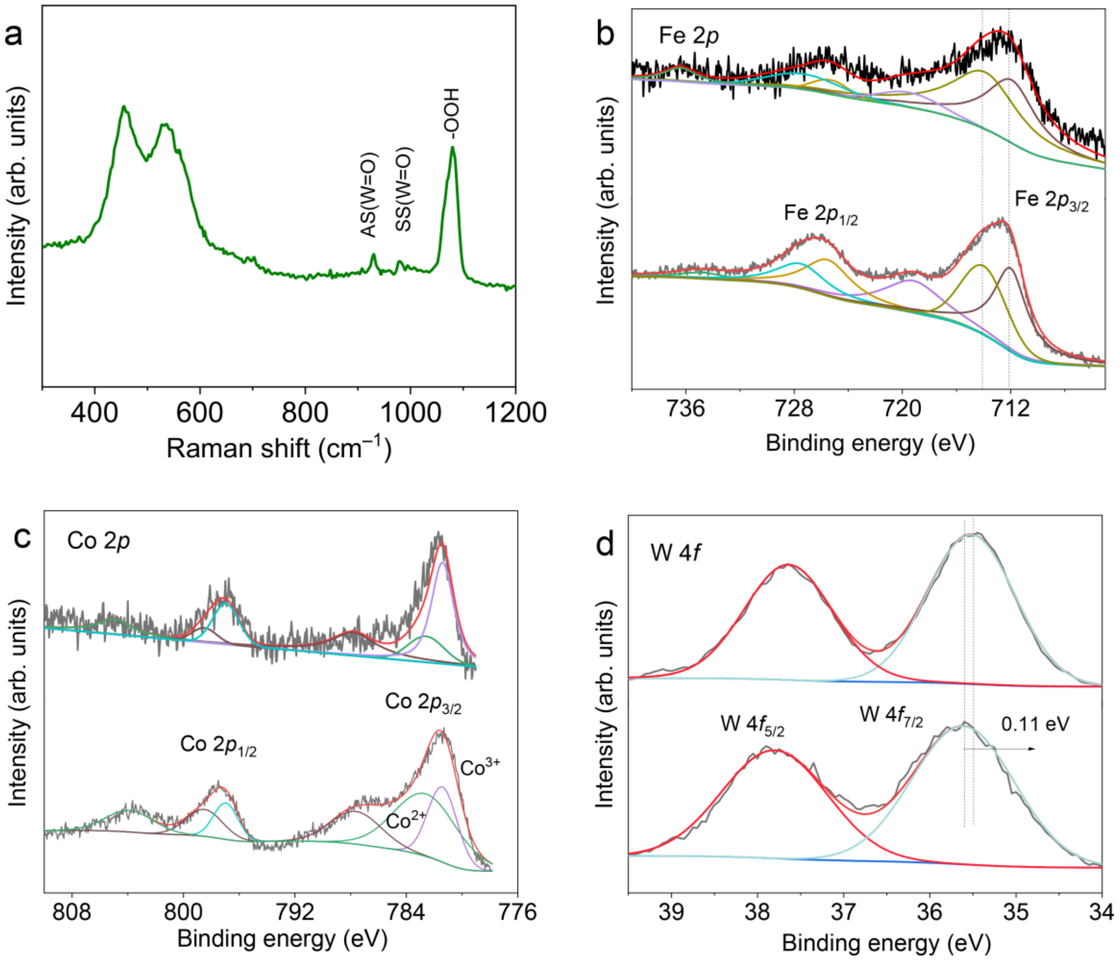

Supplementary Fig. 39 | Raman and XPS characterizations after stability tests. (**a**) Raman spectrum of PW_{12}-CoFe LDH after 1300 hours of electrolysis. Comparison of XPS spectra for PW_{12}-CoFe LDH (top, after reaction; bottom, before reaction) in the (**b**) Fe 2*p*, (**c**) Co 2*p*, and (**d**) W 4*f* regions after 1300 hours of electrolysis. Source data are provided as a Source Data file.

After 1300 hours of electrolysis, the XRD pattern of PW_{12}-CoFe LDH/NF shows that the (003) and (006) planes are retained while other diffraction peaks disappear (Supplementary Fig. 38a), indicating that the layered structure of PW_{12}-CoFe LDH is preserved. The TEM image further confirms a well-preserved nanosheet structure with visible layered features (Supplementary Fig. 38b). Strong Ni diffraction peaks are also observed, suggesting that the Ni foam substrate is well protected against chloride corrosion. The Raman spectrum shows the sustained presence of PW_{12}-POM and the formation of metal oxyhydroxides, evidenced by the enhancement of the –OOH peak (Supplementary Fig. 39a). XPS analysis reveals almost no shift or intensity change in

the Fe 2*p* region (Supplementary Fig. 39b), increased Co^{3+} content in the Co 2*p* region (Supplementary Fig. 39c), and stable W 4*f* signal intensity (Supplementary Fig. 39d). These results also indicate that PW_{12}-POM stabilizes Fe and promotes the formation of metal oxyhydroxides.

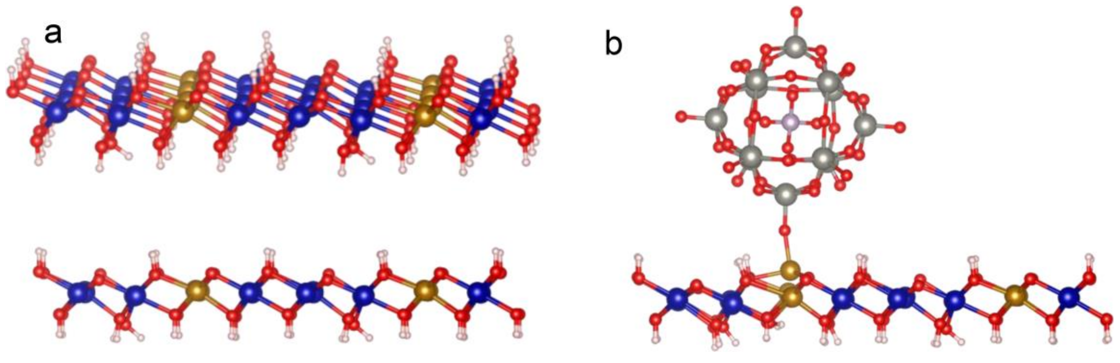

Supplementary Fig. 40 | Structural diagrams. Structural diagrams of (**a**) CoFeOOH and (**b**) PW_{12}-CoFeOOH.

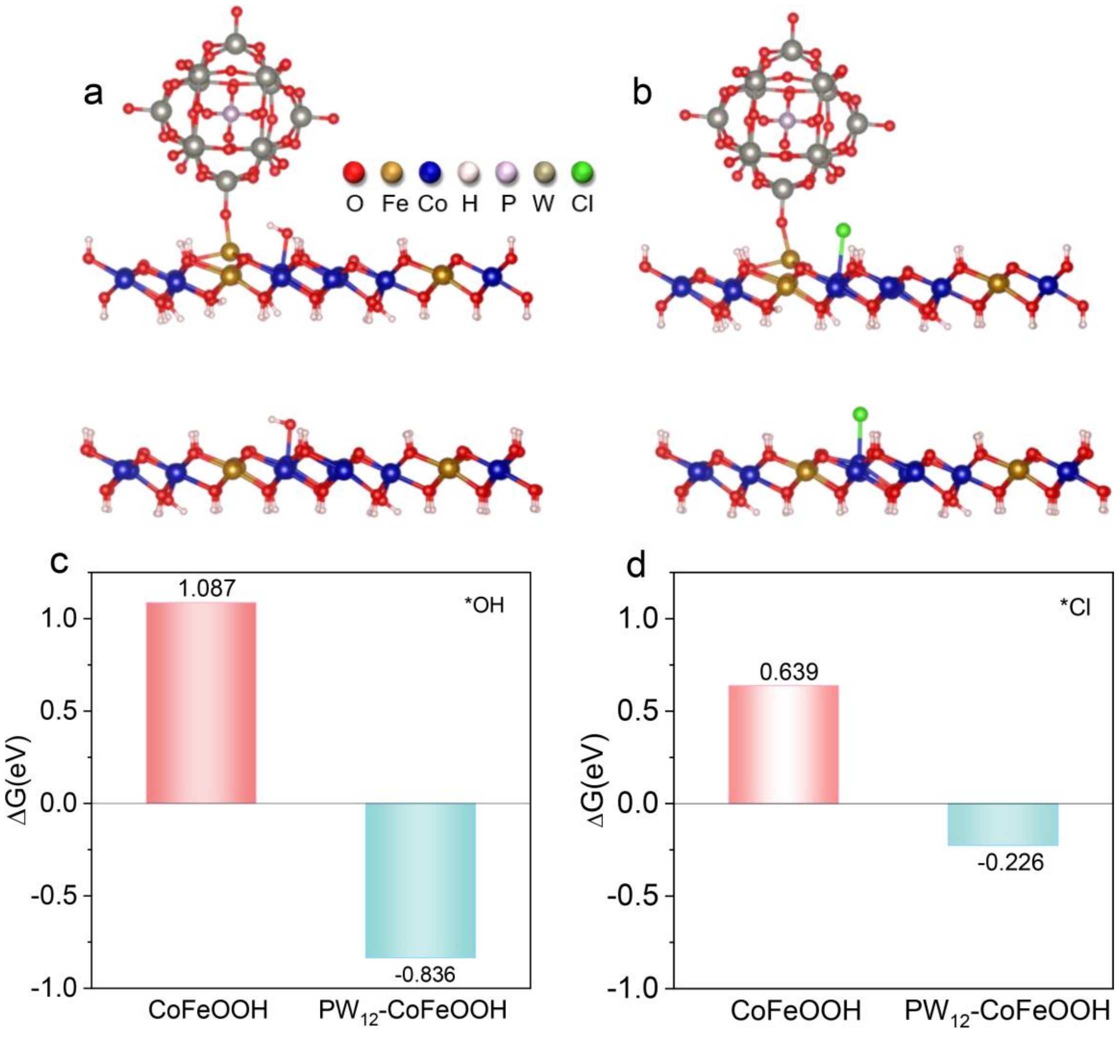

Supplementary Fig. 41 | *OH and *Cl adsorption energy changes. Optimized structures of CoFeOOH and PW_{12}-CoFeOOH with (**a**) *OH and (**b**) *Cl species adsorbed. Adsorption energy values for (**c**) *OH and (**d**) *Cl on CoFeOOH and PW_{12}-CoFeOOH.

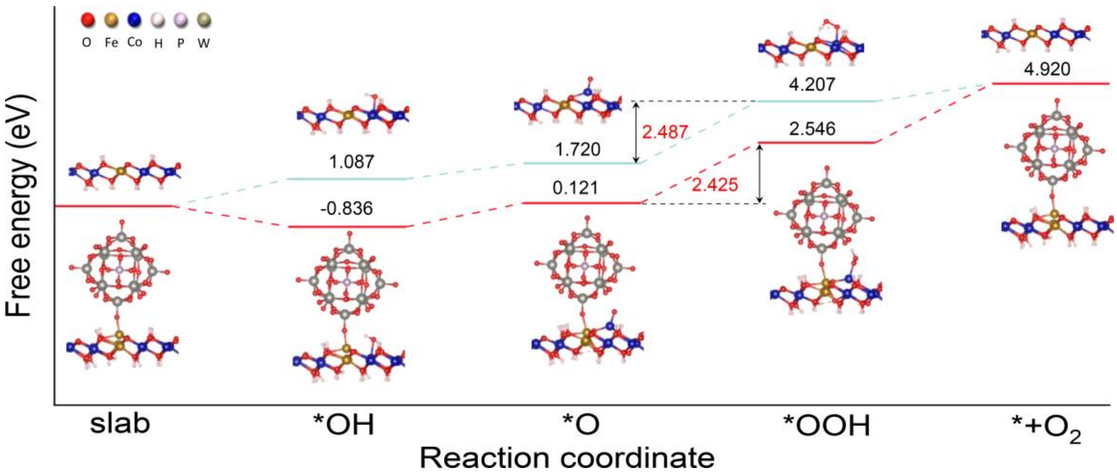

Supplementary Fig. 42 | Theoretical calculations. Gibbs free-energy profile for the four-step OER pathway on the Co site of PW_{12}-CoFeOOH versus CoFeOOH.

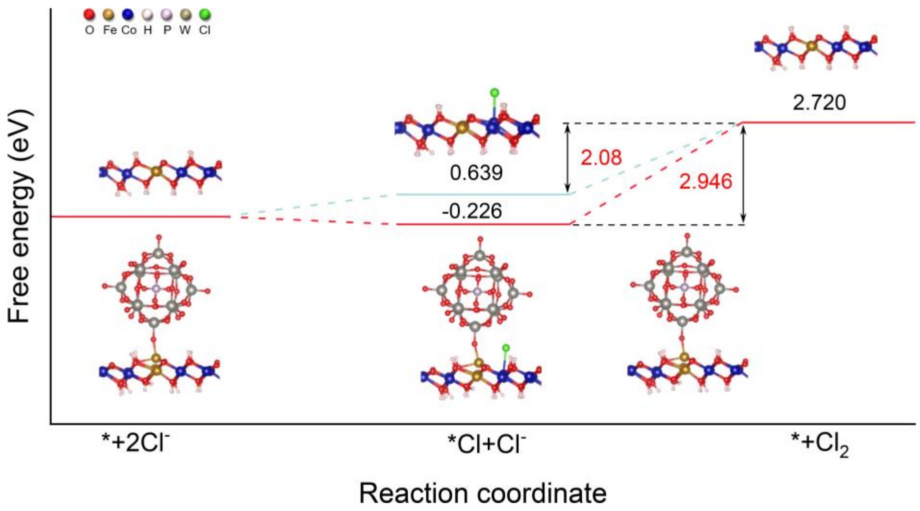

Supplementary Fig. 43 | Theoretical calculations. Gibbs free-energy profile for the two-step ClOR pathway on the Co sites of PW_{12}-CoFeOOH versus CoFeOOH.

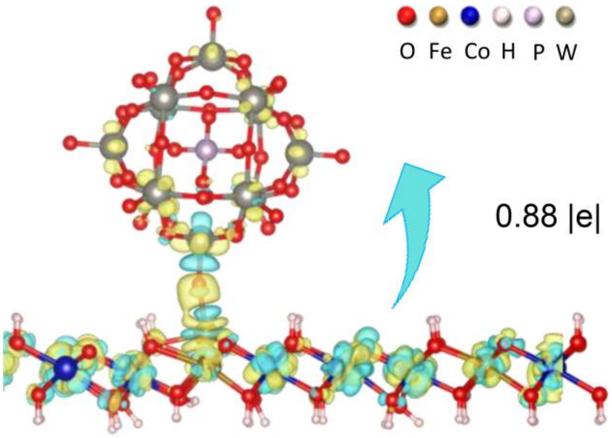

Supplementary Fig. 44 | Charge density difference diagram of PW_{12}-CoFeOOH. The yellow region represents charge accumulation and the cyan region represents charge depletion.

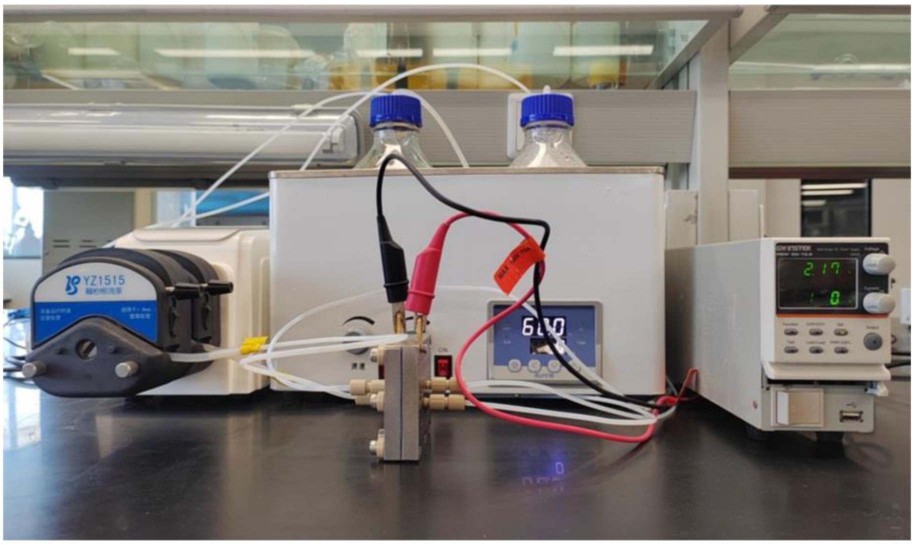

Supplementary Fig. 45 | MEA electrolyser. Photograph of the MEA electrolyser.

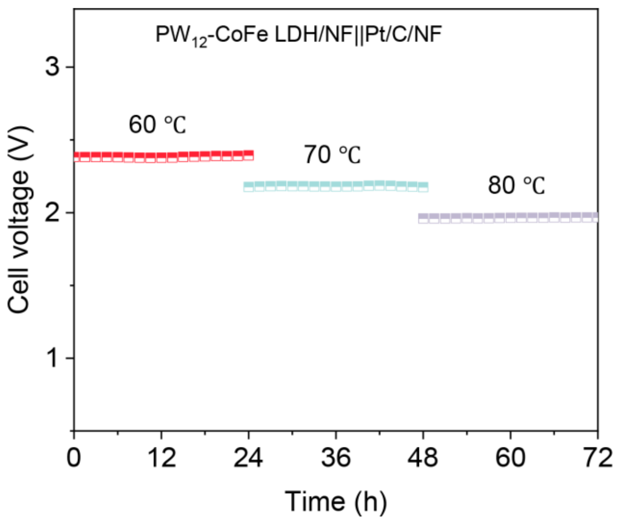

Supplementary Fig. 46 | Time-dependent potential curves of PW_{12}-CoFe LDH/NF||Pt/C/NF in alkaline seawater at different reaction temperatures without *iR* correction. Source data are provided as a Source Data file.

Supplementary Table 1. Mass ratios of elements for CoFe LDH and PW_{12}-CoFe LDH, as detected by ICP-OES.

Catalysts	Co (wt%)	Fe (wt%)	W (wt%)	P (wt%)
CoFe LDH	75.55	24.44	/	/
PW_{12}-CoFe LDH	54.10	17.46	28.04	0.36

Supplementary Table 2. Comparison of the overpotentials of the PW_{12}-CoFe LDH/NF anode with recently reported electrocatalysts in alkaline seawater.

Electrocatalysts	Electrolyte	*j* (mA cm^{-2})	Overpotential (mV)	Reference
PW_{12}-CoFe LDH/NF	1 M KOH +seawater	1000<br>500<br>100	368<br>325<br>265	This work
CoFeAl LDH/NF	20wt.% NaOH + satu. NaCl	10<br>200	256<br>~320	*Nat. Commun.* **15**, 4712 (2024)
CoFe-Ci @GQD	1 M KOH + 0.5 M NaCl	100	255	*Nat. Sustain.* **7**, 158–167 (2024)
Ir/CoFe LDH	6 M NaOH + 2.8 M NaCl	10	202	*Nat. Commun.* **15** 1973 (2024)
CoFePBA/Co_2P	1 M NaOH + 0.5 M NaCl	10<br>100	257<br>297	*Angew. Chem. Int. Ed.* **62**, e202309882 (2023)
CoFe-Ni_2P/NF	1 M KOH + seawater	100<br>500	266<br>304	*Adv. Energy Mater.* **13**, 2301475 (2023)
B-Co_2Fe LDH/NF	1 M KOH + seawater	100<br>500	310<br>376	*Nano Energy* **83**, 105838 (2021)
NiFe-CuCo LDH	6 M KOH + seawater	100<br>300<br>500	259<br>278<br>283	*Proc. Natl. Acad. Sci. USA* **119**, e2202382119 (2022)
MoO_3@CoO/CC	1 M KOH + 0.5 M NaCl	800	650	*Nat. Commun.* **15**, 2481 (2024)
S-(Ni,Fe)OOH	1 M KOH + seawater	100<br>500	300<br>398	*Energy Environ. Sci.* **13**, 3439 (2020)
(NiFe)C_2O_4/NF	1 M KOH + seawater	100<br>500<br>1000	280<br>339<br>349	*Angew. Chem. Int. Ed.* **63**, e202316522 (2024)
RuMoNi	1 M KOH + seawater	10<br>1000	245<br>430	*Nat. Commun.* **14** 3607 (2023)

Supplementary Table 3. Comparison of the stability of the PW_{12}-CoFe LDH/NF anode with recently reported electrocatalysts in alkaline seawater.

Electrocatalysts	Electrolyte	*j* (mA cm^{-2})	Stability (h)	Reference
PW_{12}-CoFe LDH/NF	1 M KOH +seawater	1000	1300	This work
		2000	600	
CoFeAl LDH/NF	20wt.% NaOH + satu. NaCl	1000	500	*Nat. Commun.* **15**, 4712 (2024)
		2000	350	
CoFe-Ci @GQD	1 M KOH + 0.5 M NaCl	1250	2800	*Nat. Sustain.* **7**, 158–167 (2024)
Ir/CoFe LDH	6 M NaOH + 2.8 M NaCl	800	1000	*Nat. Commun.* **15** 1973 (2024)
CF@CF-phy/NF	1 M KOH +seawater	1000	1000	*ACS Nano* **19**, 1530–1546 (2025)
CoFePBA/Co_2P	20wt.% NaOH + satu. NaCl	1000	1000	*Angew. Chem. Int. Ed.* **62**, e202309882 (2023)
		2000	100	
CoFe-Ni_2P/NF	1 M KOH + seawater	500	500	*Adv. Energy Mater.* **13**, 2301475 (2023)
B-Co_2Fe LDH/NF	1 M KOH + seawater	500	100	*Nano Energy* **83**, 105838 (2021)
Cr-CoFe LDH/NF	1 M KOH + seawater	500	100	*Small* **20**, 2307294 (2024)
FCDs/FeCoSe-VSe/NF	1 M KOH + seawater	200	200	*Appl. Surf. Sci.* **680**, 161456 (2025)
CeO_{2-x}@CoFe LDH/NF	1 M KOH + 0.5 M NaCl	50	35	*Inorg. Chem. Front.* **7**, 4461–4468 (2020)
$CoCO_3$/CoFe LDH/NF	1 M KOH + seawater	1000	1000	*Small* **21**, 2409627 (2025)
RuCo-$CoFe_2O_4$@IF	1 M KOH + seawater	1000	150	*Chem. Eng. J.* **503**, 158346 (2025)
NiFe-CuCo LDH	6 M KOH + seawater	500	500	*Proc. Natl. Acad. Sci. USA* **119**, e2202382119 (2022)
MoO_3@CoO/CC	1 M KOH + seawater	1000	600	*Nat. Commun.* **15**, 2481 (2024)
RuMoNi	1 M KOH + seawater	500	3000	*Nat. Commun.* **14**, 3607 (2023)
S-(Ni,Fe)OOH	1 M KOH + seawater	100	100	*Energy Environ. Sci.* **13**, 3439 (2020)
NiMoN@NiFeN/NF	1 M KOH + seawater	500	100	*Nat. Commun.* **10**, 5106 (2019)

$(NiFe)C_2O_4/NF$	1 M KOH + seawater	1000	600	*Angew. Chem. Int. Ed*. **63**, e202316522 (2024)
OP-NiCo LDH	1 M KOH + 0.5 M NaCl	100	500	*Appl. Catal. B-Environ. Energy* **332**, 122749 (2023)
NiFe LDH@Ag	1 M NaOH + seawater	400	5000	*Adv. Mater.* **36**, 2306062 (2024)

Note: GQD, graphene quantum dots; phy, phytate; CF, CoFe LDH; FCDs, fluorine-doped carbon dots; IF, iron foam.

Supplementary Table 4. Co and Fe amounts in electrolyte of CoFe LDH/NF anode in alkaline seawater, detected by ICP-OES at different times during ASO.

Time (h)	Co (ppm)	Fe (ppm)
1	2.11	1.06
10	6.32	2.11
20	9.83	3.17
30	13.11	4.58
40	14.98	5.15
50	17.09	6.06
60	19.66	6.34
80	21.30	6.91

Supplementary Table 5. Co, Fe, W, and P amounts in electrolyte of PW_{12}-CoFe LDH/NF anode in alkaline seawater, detected by ICP-OES at different times during ASO.

Time (h)	Co (ppb)	Fe (ppb)	W (ppb)	P (ppb)
1	47.28	15.68	2.00	0.14
10	118.20	20.90	2.13	0.15
20	141.84	21.34	2.16	0.15
30	148.16	21.69	2.14	0.16
40	147.51	21.78	2.12	0.16
50	149.88	22.04	2.16	0.16
60	150.82	22.12	2.09	0.16
80	148.69	21.95	2.14	0.16

Supplementary Table 6. Comparison of cell voltage and tolerance of PW_{12}-CoFe LDH/NF‖Pt/C/NF with reported the two-electrode system.

Two-electrode system	Electrolyte	Temperature (°C)	Cell voltage (V)/A cm^{-2}	Tolerance (h)/A cm^{-2}	Reference
PW_{12}-CoFe LDH/NF‖Pt/C/NF	1 M KOH + seawater	60	2.39/1	1000/1	This work
RuO_2/NF‖Pt/C/NF	1 M KOH + seawater	60	3.18/0.8	/	This work
NiFeP/NF‖NiCoP–Cr_2O_3/NF	1 M KOH + seawater	60	~1.9/1	1000/1	*Nature* **639**, 360–367 (2025)
CoFe-Ci@GQDs/NF(+, -)	1 M KOH + 0.5 M NaCl	30 suns	2.6/0.446	200/0.446	*Nat. Sustain.* **7**, 158–167 (2024)
CoFeAl LDH/NF‖NF	20wt.% NaOH + satu. NaCl	80	2.06/1	500/1	*Nat. Commun.* **15**, 4712 (2024)
MoO_3@CoO/CC (+, -)	1 M KOH + seawater	Room temperature	1.93/1	500/1	*Nat. Commun.* **15**, 2481 (2024)
RuMoNi (+, -)	1 M KOH + seawater	40	1.73/1	240/0.5	*Nat. Commun.* **14**, 3607 (2023)
CF@CF-phy/NF‖Pt/C/NF	1 M KOH + seawater	Room temperature	2.5/0.5	200/0.5	*ACS Nano* **19**, 1530–1546 (2025)
CoFe-Ni_2P/NF(+, -)	6 M KOH + seawater	Room temperature	2.25/1	350/1	*Adv. Energy Mater.* **13**, 2301475 (2023)
$CoCO_3$/CoFe LDH/F‖Pt/C/NF	1 M KOH + seawater	Room temperature	~2.45/0.5	200/0.5	*Small* **21**, 2409627 (2025)
CoS_2@CoFe LDH/NF (+, -)	1 M KOH + seawater	Room temperature	1.96/0.1	18/0.1	*Small* **21**, 2406431 (2025)
RuCo-$CoFe_2O_4$@IF (+, -)	1 M KOH + seawater	30	1.73/0.1	600/0.1	*Chem. Eng. J.* **503**, 158346 (2025)
