# Supplementary material for: Engineered PW12-polyoxometalate docked Fe sites on CoFe hydroxide anode for durable seawater electrolysis
Source: Nat Commun. 2025 Jul 1;16:5541. doi: 10.1038/s41467-025-60620-9 (PMC12214576; doi:10.1038/s41467-025-60620-9)
Supplement: Supplementary file 2 — Description of Additional Supplementary Files [file 41467_2025_60620_MOESM2_ESM.pdf]

Description of Additional Supplementary Files

File name: **Supplementary Data 1**

Description: The optimized CONTCAR for PW_{12}-CoFe LDH.
